# Acquired drug resistance interferes with the susceptibility of prostate cancer cells to metabolic stress

**DOI:** 10.1186/s11658-022-00400-1

**Published:** 2022-11-18

**Authors:** Jessica Catapano, Marcin Luty, Tomasz Wróbel, Maciej Pudełek, Katarzyna Piwowarczyk, Sylwia Kędracka-Krok, Maciej Siedlar, Zbigniew Madeja, Jarosław Czyż

**Affiliations:** 1 Department of Cell Biology, Faculty of Biochemistry, Biophysics and Biotechnology, Jagiellonian University, Gronostajowa 7, 30-387 Kraków, Poland; 2 Department of Physical Biochemistry, Faculty of Biochemistry, Biophysics and Biotechnology, Jagiellonian University, Gronostajowa 7, 30-387 Kraków, Poland; 3 Department of Clinical Immunology, Institute of Pediatrics, Faculty of Medicine, Jagiellonian University Medical College, Wielicka 265, 30-663 Kraków, Poland; 4 Proteomics and Mass Spectrometry Laboratory, Malopolska Centre of Biotechnology, Jagiellonian University, Gronostajowa 7A, 30-387 Kraków, Poland

**Keywords:** Metabolic stress, Drug resistance, Prostate cancer, Metabolic adaptation, Metformin

## Abstract

**Background:**

Metformin is an inhibitor of oxidative phosphorylation that displays an array of anticancer activities. The interference of metformin with the activity of multi-drug resistance systems in cancer cells has been reported. However, the consequences of the acquired chemoresistance for the adaptative responses of cancer cells to metformin-induced stress and for their phenotypic evolution remain unaddressed.

**Methods:**

Using a range of phenotypic and metabolic assays, we assessed the sensitivity of human prostate cancer PC-3 and DU145 cells, and their drug-resistant lineages (PC-3_DCX20 and DU145_DCX20), to combined docetaxel/metformin stress. Their adaptation responses have been assessed, in particular the shifts in their metabolic profile and invasiveness.

**Results:**

Metformin increased the sensitivity of PC-3 wild-type (WT) cells to docetaxel, as illustrated by the attenuation of their motility, proliferation, and viability after the combined drug application. These effects correlated with the accumulation of energy carriers (NAD(P)H and ATP) and with the inactivation of ABC drug transporters in docetaxel/metformin-treated PC-3 WT cells. Both PC-3 WT and PC-3_DCX20 reacted to metformin with the Warburg effect; however, PC-3_DCX20 cells were considerably less susceptible to the cytostatic/misbalancing effects of metformin. Concomitantly, an epithelial–mesenchymal transition and Cx43 upregulation was seen in these cells, but not in other more docetaxel/metformin-sensitive DU145_DCX20 populations. Stronger cytostatic effects of the combined fenofibrate/docetaxel treatment confirmed that the fine-tuning of the balance between energy supply and expenditure determines cellular welfare under metabolic stress.

**Conclusions:**

Collectively, our data identify the mechanisms that underlie the limited potential of metformin for the chemotherapy of drug-resistant tumors. Metformin can enhance the sensitivity of cancer cells to chemotherapy by inducing their metabolic decoupling/imbalance. However, the acquired chemoresistance of cancer cells impairs this effect, facilitates cellular adaptation to metabolic stress, and prompts the invasive front formation.

**Supplementary Information:**

The online version contains supplementary material available at 10.1186/s11658-022-00400-1.

## Background

Drug resistance is determined by the efficiency of cellular self-defense systems that participate in the intracellular repair processes, drug metabolism, and drug efflux. These systems reduce the sensitivity of the cells to chemotherapeutics, toxins and/or remove the drugs from the cells [1, 2]. Drug resistance represents a serious challenge for the current oncology because it prompts the application of increasing doses of the drugs to counteract tumor growth [3, 4]. Its consequences are especially dreadful for patients with prostate cancer who, owing to their prevalently advanced age [5], are particularly susceptible to adverse effects of chemotherapy. Elaboration of new treatment strategies, which would be directly or indirectly targeted at the activity of cellular self-defense systems (including cell cycle-related and anti-apoptotic proteins, DNA-repair systems, autophagy, and transmembrane transporters), is a potential way to overcome cancer drug resistance [3, 6]. Recently, metabolic therapies have been suggested to limit the growth and progression of aggressive tumors. Among them, attempts to introduce nutritional deprivation and metabolic blockers into cancer treatment have now been carried out worldwide. For instance, dietary restriction of essential amino acids (including arginine and methionine) can give promising clinical outcome, when combined with other anticancer approaches [7–9]. In turn, metabolic blockers can impair drug-resistance systems of prostate cancer cells and increase the chemosensitivity of prostate tumors, thus reducing the effective doses and side effects of cytostatic drugs [10, 11]. On the other hand, the consequences of metabolic block for prostate cancer microevolution and its progression remain largely unaddressed and require careful investigation.

Interference of metabolic blockers with the tumor drug-resistance systems relies on the impairment of energy supply for ABC (ATP-binding cassette) transporter proteins [12]. They represent a heterogeneous group of membrane proteins that remove xenobiotics from the cells in an ATP-dependent manner. The expression levels, membrane localization, and activity of ABC pumps are enhanced under chemotherapeutic stress. In turn, the inhibitors of ATP production impair ABC pumps’ function in prostate cancer cells. For instance, fenofibrate (a standard anti-hyperlipidemic drug [13]) has been shown to sensitize prostate cancer cells to docetaxel through the interference with their energy metabolism and ATP/ABC-dependent drug efflux [14]. Similar activity was reported for OBP-51602 [15] and metformin, which inhibits mitochondrial complex I, impairs the function of ABC systems, and augments the cytostatic action of chemotherapeutics in vitro and in vivo [16–18]. On the other hand, the efficiency of metformin-based treatment in the therapy of drug-resistant prostate tumors has been questioned by reports on its relatively negligible anticancer potential [19, 20]. These conflicting data prompted us to hypothesize that the cytostatic effects of metformin can be counterbalanced by the metabolic adaptation of drug-resistant cells [21].

Cancer cells can counteract chemotherapeutic stress and compensate for the increased ATP demand from efflux pumps by temporary and/or permanent metabolic reprogramming [15, 22, 23]. Consequently, they efficiently exploit microenvironmental resources and/or adapt to the impairment of energy supply by metabolic blockers. Recently, the switch from anaerobic to aerobic metabolism has been reported to correlate with the acquired chemoresistance of prostate cancer cells [15, 17]. It helps to provide ATP resources for overactive efflux pumps in cancer cells, but also sensitizes the cells to the blockers of mitochondrial respiration. Consequently, this increases the potential of metronomic approaches based on the oxidative phosphorylation (OXPHOS) inhibitors in the treatment of advanced (malignant) tumors. On the other hand, cellular adaptation to metabolic stress, for instance due to metabolic elasticity [24, 25], may represent an important (although underestimated) factor that facilitates the survival of cancer cells in harmful microenvironments [26]. In particular, the switch from OXPHOS to aerobic glycolysis (Warburg effect) can help the cells to retain drug resistance in the presence of metabolic blockers. Owing to the links between metabolic profile and cancer cell invasiveness [25, 27], this process may facilitate the microevolution of invasive cell lineages, with dreadful consequences of this process for the patients. However, the links between the acquired drug resistance, metabolic profile of prostate cancer cells, and their adaptability to the metabolic stress largely remain to be identified.

We have previously shown that fenofibrate increases the sensitivity of drug-resistant prostate cancer cell lineages to chemotherapeutics [14]. Concomitantly, we observed an array of self-defense responses of prostate cancer cells that survive the initial chemotherapeutic/metabolic shock [28]. Similarly, metformin was shown to interfere with the acquired drug resistance of prostate cancer cells [17]. However, the effect of acquired drug resistance on cellular adaptation to metformin-induced metabolic block still remains to be comprehensively scrutinized. We fill this gap by focusing on these reactions and addressing their effect on the efficiency of metformin in the combined metabolic therapies of prostate tumors. In particular, we scrutinized the interrelations between the drug resistance of prostate cancer cells and their sensitivity to the combined docetaxel/metformin treatment. Then, we identified the processes that underlie the sensitivity/adaptation of prostate cancer cells to the combined application of docetaxel and metformin, with a special emphasis on the role of metabolic balance in these processes. Finally, we estimated the consequences of cell adaptation to metformin-induced metabolic stress for the invasive potential of prostate cancer cells.

## Materials and methods

### Cell cultures

Human prostate carcinoma PC-3 (ATCC CRL-1435), DU145 (ATCC; HTB-81) cells, and their drug-resistant sublineages (PC-3_DCX20 and DU145_DCX20) were cultured in DMEM/F12 HAM medium (Sigma-Aldrich, St. Louis, MO, USA) with supplements (10% FBS and antibiotics). For endpoint experiments, media supplemented with docetaxel (DCX), metformin (MET), and fenofibrate (FF) were added to the cell cultures at the concentrations and timepoints indicated in the text [DCX, 0.25–20 nM; MET, 2.5–10 mM; FF, 5–25 μM (all from Sigma-Aldrich)]. These concentrations secure their specific action corresponding to the in vivo situation. For the establishment of DCX-resistant sublineages, PC-3 and DU145 cells were subjected to consecutive DCX treatment and recovery cycles, based on the exposition to DCX administered at increasing concentrations (1, 2, 5, 10, 20 nM) and recovery in the PC-3/DU145-conditioned medium [14]. The established lineages were cultivated in the standard medium for approximately 15 generation times (five passages at 1:8). Stability of acquired resistance was assessed after freezing, thawing, and drug withdrawal throughout this period.

### Proliferation and apoptosis assay

Cells were seeded into 24-well plates (Corning) at a density of 5 × 10^3^ cells/cm^2^, and cultivated for 24 h before the application of DCX/MET/FF-containing media. After 48 h, the cells were harvested and resuspended in the original culture medium. Counting was performed with a Coulter Z2 Counter (Beckman Coulter Inc., Fullerton, CA, USA). For the analyses of cell apoptosis, cells were exposed to the tested agents, harvested, resuspended in original medium, and subjected to AnnexinV/propidium iodide staining solution according to the manufacturer’s protocol (BD Biosciences [29];). Flow cytometric detection of apoptotic cells was performed with a FACSAria FACS system (Becton–Dickinson, Heidelberg, Germany). At least 5 × 10^4^ cells were analyzed for each condition.

### Cell motility and transmigration assays

Cells were seeded at the density of 5000 cells/cm^2^ (short-term incubation variant) or 1000 cells/cm^2^ (long-term incubation variant) and cultivated for 24 h. Then, the agents were added along with the medium, and the cells were analyzed immediately afterwards or after 48 h of incubation. Cell movement was estimated with the time-lapse Leica DMI6000B videomicroscopy system equipped with a temperature chamber (37 ± 0.2 °C)/(5% CO_2_), IMC contrast optics, and a cooled, digital DFC360FX CCD camera. Cell trajectories were constructed from the sequences of centroid positions recorded for 8 h at 300 s time intervals (with a ×10, NA 0.75 objective) to calculate single cell movement parameters: total length of single cell trajectory (μm), speed of cell movement (total length of single cell trajectory/time of registration (μm/min), total length of single cell displacement (displacement; μm), and speed of cell displacement (total length of single cell displacement/time of registration (μm/min). Data from > 50 trajectories (more than three independent experiments) were then pooled and analyzed to calculate cell movement parameters at the population level and to perform statistical analysis [30]. For the transmigration assays, cells were seeded on the microporous membranes (Transwell; 8 μm) at a density of 2 × 10^4^ cells per well and grown for 24 h before the administration of the analyzed agents and further cultivation of the cells for the next 96 h. Then, the cells that managed to transmigrate the membranes were harvested and counted to calculate the transmembrane penetration index (%) [31, 32].

### Metabolic assays

MTT assay was performed to estimate intracellular NAD(P)H contents. Cells were seeded into 96-well plates (Eppendorf) at a density of 5 × 10^3^ cells per well, incubated for 24 h, treated with DCX/MET/FF for 48 h, and exposed to thiazolyl blue tetrazolium bromide (0.5 mg/ml in water, Sigma-Aldrich; no. M5655) for 2 h at 37 °C. Afterwards, formazan was dissolved in isopropanol, and its *A*_570_ absorbance was measured with Multiskan FC Microplate Reader (Thermo Fisher Scientific). Intracellular ATP contents were estimated using the luminescence ATPlite detection assay system (6016947; Perkin-Elmer, Warszawa, Poland). The cells were cultivated in the presence of DCX/MET/FF for 48 h and lysed, and the cell lysates were transferred to white plates for the collection of luminescence signals with the Infinite M200 reader. Formazan/ATP contents per sample (population level) or per 10^5^ cells (referring to averaged NAD(P)H/ATP content in single cells) were calculated from calibration curves. For lactate secretion analyses, the cells were prepared as described above. Intracellular lactate contents were performed using a lactate assay kit (cat. no. MAK064; Sigma-Aldrich) according to the manufacturer’s protocol. The cells were plated in 96-well plates, incubated with DCX/MET/FF for 48 h and lysed. The lysates were incubated with reagents delivered by the manufacturer. *A*_570_ absorbance was measured with Multiskan FC Microplate Reader. Lactate levels per sample (population level) or per 10^5^ cells (referring to averaged lactate content in single cells) were calculated from calibration curves.

### Immunofluorescence

Immunostaining was performed on formaldehyde/Triton X-100-fixed/permeabilized cells [formaldehyde (FA): 3.7%, 20 min at room temperature (RT); Triton X-100: 0.1%, 10 min in RT]. After incubation in the presence of 3% BSA, primary antibodies [mouse anti-vinculin IgG (no. V9131, Sigma-Aldrich), rabbit anti-Cx43 IgG (no. C6219, Sigma-Aldrich), goat anti-Snail-1 N-terminal IgG (no. SAB2501370, Sigma-Aldrich)] were applied for 1 h. Afterwards, the cells were labeled with Alexa Fluor 488-conjugated goat anti-mouse IgG and chicken anti-goat IgG (no. A11001, Thermo Fisher Scientific), and counterstained with TRITC-conjugated phalloidin (nos. 49409 and 77418, Sigma-Aldrich), Alexa Fluor 647-conjugated chicken anti-rabbit IgG (no. A21443, Thermo Fisher Scientific), and Hoechst 33258 (no. B2883, Sigma-Aldrich) [33, 34]. Image acquisition and processing was performed with a Leica DMI6000B microscope (Leica Microsystems, Wetzlar, Germany) equipped with the Total Internal Reflection Fluorescence (TIRF) and Nomarski Interference Contrast (DIC) modules. The 40× NA 1.47 oil immersion objective and 14-bit Hamamatsu 9100-02 EM-CCD camera were controlled/processed by LASX  (Leica) operation/deconvolution and ImageJ software.

### Calcein efflux assay

For the estimation of drug-efflux efficiency, cells were washed twice with warm DPBS before medium replacement for FluoroBrite DMEM (supplemented with 10% FBS and 1% GlutaMAX). Calcein-AM (Invitrogen, no. C3099) was administrated at a concentration of 1 µg/ml in the FluoroBrite DMEM. Kinetics of calcein fluorescence intensity that illustrates its efflux were monitored with Leica DMI6000B fluorescence microscope (see above) immediately after calcein-AM administration, using Alexa 488 filter set (excitation—BP 470/40 nm; emission—BP 525/50) and time-lapse imaging module (time step, 5 min; total acquisition time, 60 min). Obtained images were processed with ImageJ software. For the fluorometric analyses of calcein-stained specimens, stacks of fluorescence images of at least 16 randomly chosen confluent culture regions were collected. In each experiment, the stacks were obtained with the same excitation/exposure settings (excitation/camera gain/time of exposition). Fluorescence index was estimated for each stack with LasX software (Leica) and calculated for each specimen [14].

### Metabolic profile

Cells were plated in Seahorse XF eight-well plates 48/72 h before the measurement, subjected to DCX/MET/FF for 24/48 h, and incubated in Seahorse XF Assay Media at 37 °C for 1 h without CO_2_ immediately before starting the Real-Time ATP Rate Assay (Seahorse Bioscience; 1 µM oligomycin; 1 µM/0.5 µM rotenone/antimycin A). Oxygen consumption rate (OCR) and extracellular acidification rate (ECAR) measurements were performed and analyzed with the Seahorse Analyzer XF HS Mini/XFp software and normalized to cell number.

### Immunoblotting

For the estimation of total Cx43 levels, the cells were seeded into 75 cm^2^ Falcon dishes at the density of 5 × 10^5^ cells per dish and incubated up to 70–80% confluency before the application of DCX/MET for 48 h. Then, the cells were directly harvested, centrifuged, and dissolved in lysis buffer with protease inhibitor cocktail, followed by their freeze–thawing and sonication, and estimation of the total protein content by Bradford assay. Protein samples (20 µg) were subjected to SDS–PAGE electrophoresis on 12% polyacrylamide gel (Laemmli protocol) and transferred to PVDF membranes (Immun-Blot PVDF Membrane, #1620177; Bio-Rad, Hercules, CA). Blocking of unspecific staining (skimmed milk/TBST solution) was followed by the application of primary monoclonal rabbit anti-Cx43 (no. C6219; 1:3000, Sigma-Aldrich), washing, and administration of HRP conjugated goat anti-rabbit IgG (no. 31466, Thermo Fisher Scientific). Signal detection was performed with the chemiluminescent HRP substrate Luminata Crescendo (no. WBLUR0500, Merck) and membrane imaging MicroChemi system (DNR Bio-Imaging System).

### Calcein transfer assay

Donor and acceptor cells were incubated in the presence of DCX/MET for 24 h. Donor cells were then stained with calcein and DiI (Life Technologies; C3099; 5 μM and 10 μM) as described previously [35] and seeded onto the monolayers of acceptor cells at 1:50 ratio. After 1 h of coincubation of donor and acceptor cells, a transfer of calcein from at least 200 donor cells per coverslip was analyzed using a Leica DMI6000B inverted fluorescence microscope (Leica Alexa 488 filter set) equipped with LasX software. It was further quantified as the percentage of donor cells, which successfully coupled with an acceptor monolayer (coupling index; *c*_i_) and averaged number of coupled acceptor cells/a donor cell (coupling ratio; *c*_r_).

### Tandem mass spectrometry

LC–MS/MS was performed with the reversed-phase liquid chromatography (RP-LC) system (UltiMate 3000 RSLCnano System, Thermo Fisher Scientific) coupled to Q-Exactive mass spectrometer (Thermo Scientific). The filter-assisted sample preparation (FASP) method was used to prepare cell lysates for shotgun LC–MS/MS measurements. The peptide samples were loaded onto a trap column (Acclaim PepMap 100 C18, Thermo Fisher Scientific) and separated on an analytical column (Acclaim PepMap RSLC C18, Thermo Fisher Scientific). Eluting peptides were ionized with the Digital PicoView 550 nanospray source (New Objective) and acquired using Top12 method in an MS data-dependent mode. The LC–MS/MS data were analyzed using Proteome Discoverer 1.4 and a MASCOT server against the Swissprot_201802 database. Search result validation was performed using Percolator algorithm [36].

### Statistical analysis

All data were expressed as mean ± standard error of the mean (SEM) from at least three independent experiments (*N* ≥ 3). Statistical significance was tested with Student’s *t*-test or one-way analysis of variance(ANOVA) followed by post hoc Tukey’s comparison for variables with normal and non-normal distribution, respectively. *P* < 0.05 was considered statistically significant.

## Results

### Metformin and DCX exert additive cytostatic effects in prostate cancer populations

**Fig. 1:**
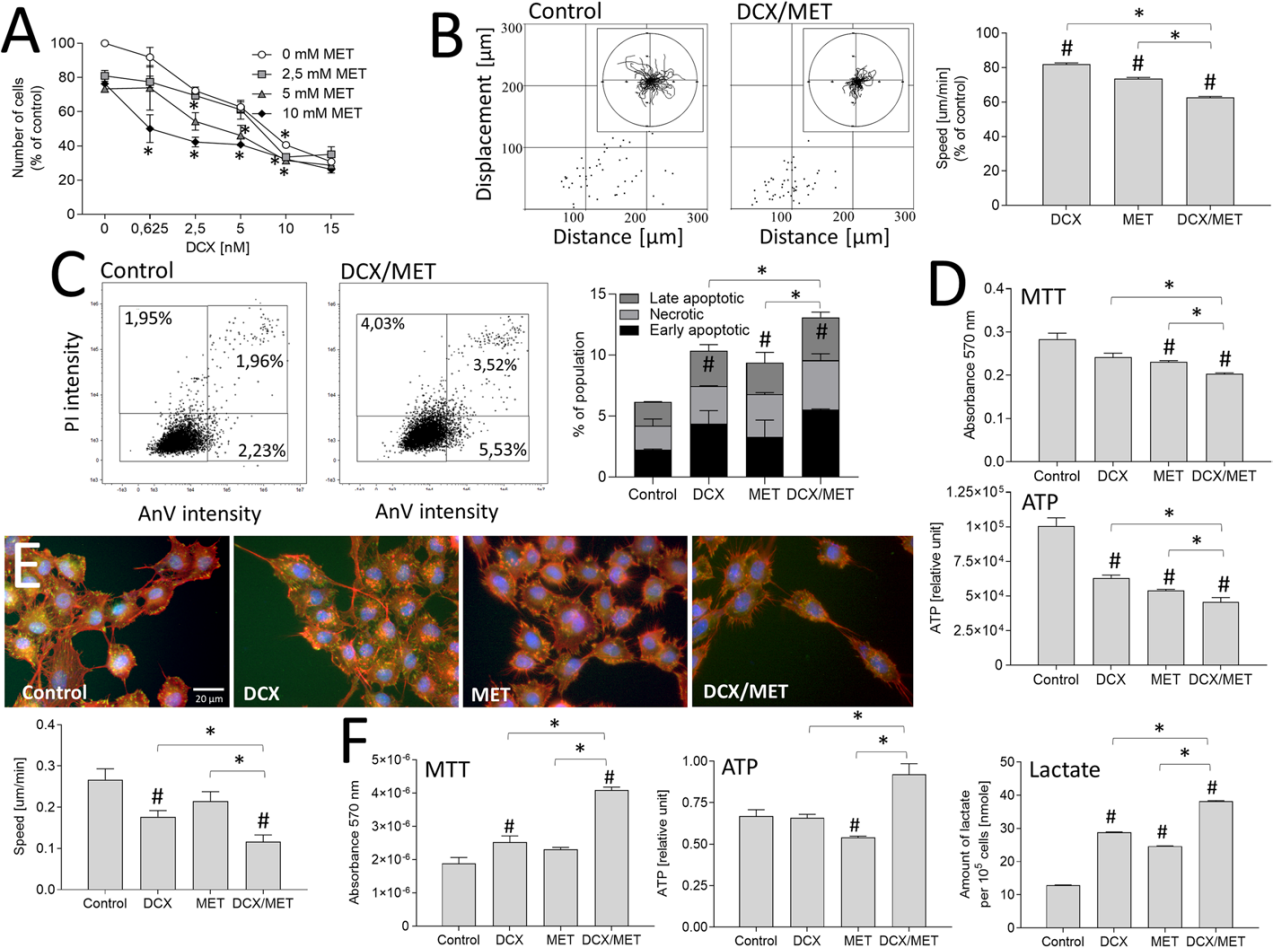
Metformin increases the sensitivity of prostate cancer cells to docetaxel. **A** PC-3 cells were cultivated in the presence of DCX (0.625–15 nM) and/or MET (2.5–10 mM) for 48 h, harvested, and counted with Coulter counter. **B** PC-3 WT motility was estimated directly after DCX (2.5 nM) and/or MET (10 mM) administration with time-lapse videomicroscopy. Cell trajectories are depicted as circular diagrams (axis scale in micrometers) drawn with the initial point of each trajectory placed at the plot origin (registered for 8 h; *N* > 50). Dot plots and column charts show movement parameters at the single-cell and population level, respectively (plotted as percentage of control; cf. Additional file 1: Fig. S1A). **C** Apoptotic response of PC-3 WT cells estimated after the long-term DCX and/or MET treatment (2.5 nM/10 mM) with annexinV/PI assay. Compensated dot plots comprise 50,000 events, classified on the basis of their bright-field ratio (cf. Additional file 1: Fig. S1B). **D** Effect of the long-term (48 h) DCX/MET (2.5 nM/10 mM) treatment on PC-3 viability, estimated at the population level with MTT (upper panel) and ATP assay (lower panel). **E** Cytoskeleton architecture (F-actin: red, vinculin: green, DNA: blue) and motility estimated in long-term (48 h) DCX and/or MET (2.5 nM/10 mM) treated PC-3 WT cells (cf. Additional file 1: Fig. S1C). **F** Metabolic profile after the long-term (48 h) DCX/MET (2.5 nM/10 mM) treatment estimated with MTT (left), ATP (middle), and lactate assay (right) and calculated per 10^5^ cells. The statistical significance of the differences was tested with Student’s *t*-test (in **A**, **C**, **D**, **F**), or by one-way ANOVA followed by post hoc Tukey’s HSD (in **B**, **E**). ^#^*P* ≤ 0.05 versus untreated control; *P ≤ 0.05 as indicated in the charts or 0 mM MET in **A**. All results are representative of at least three independent experiments (*N* ≥ 3). Error bars represent SEM. Note that MET increases DCX sensitivity, NAD(P)H/ATP levels, and lactate production in PC-3 cells

Analyses of the additive cytostatic effects of docetaxel (DCX) and metformin (MET) in human prostate cancer PC-3 populations revealed that both agents inhibited PC-3 proliferation in the range of tested concentrations. Moreover, metformin (2.5–10 mM) considerably augmented PC-3 sensitivity to DCX (0.625–15 nM; Fig. 1A). Accordingly, time-lapse analyses demonstrated an immediate inhibitory effect of DCX (2.5 nM) on PC-3 WT cell motility, which was again augmented by concomitant application of 10 mM MET (approximately 40% inhibition compared with control; Fig. 1B, cf. Additional file 1: Fig. S1A). This short-term effect was followed by an apoptotic response of PC-3 cells, which was most prominent in the presence of DCX/MET (Fig. 1C; cf. Additional file 1: Fig. S1B). It was correlated with a less pronounced (although significant) decrease of intracellular NAD(P)H and ATP contents in DCX/MET-treated PC-3 populations (Fig. 1D). However, visualization of PC-3 morphology and cytoskeletal architecture revealed a considerable fraction of cells apparently resistant to the 48-h-long combined DCX/MET stress. They displayed a strongly inhibited motility (Fig. 1E, lower panel; cf. Additional file 1: Fig. S1C), but considerably increased averaged NAD(P)H (as illustrated by MTT assay), ATP, and lactate contents per cell (Fig. 1F). Corresponding assays demonstrated the synergy of the cytostatic effects of DCX/MET in DU145 populations (cf. Additional file 1: Fig. S2). These data indicate that metformin increases the sensitivity of prostate cancer cells to docetaxel and prompts the selective survival of stress-resistant cells.

### Acquired chemoresistance of PC-3 cells reduces their susceptibility to the combined DCX/MET treatment

**Fig. 2:**
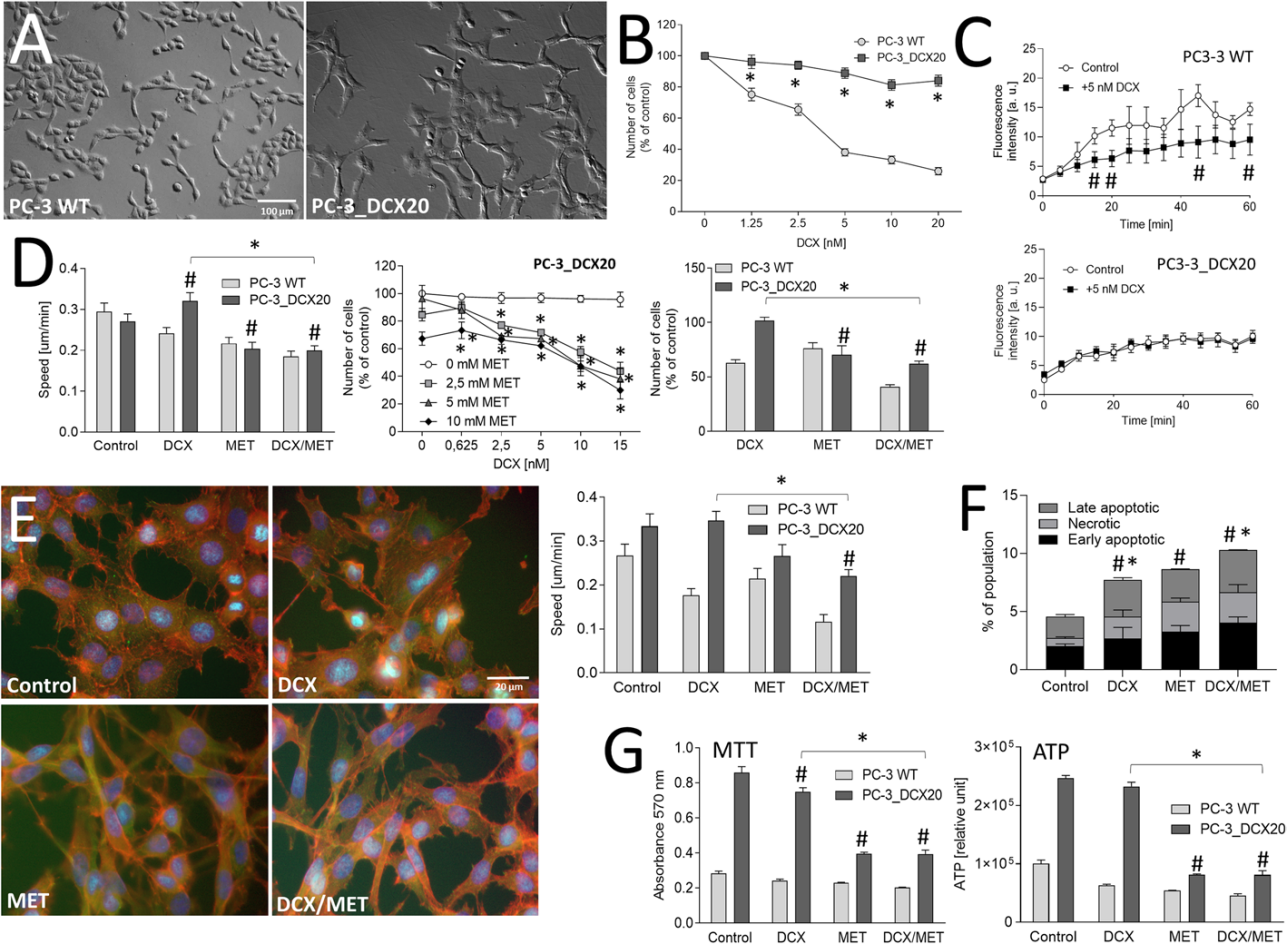
Drug resistance attenuates the reactivity of prostate cancer cells to the combined DCX/MET stress. **A** Morphology of PC-3_DCX20 cells. **B** DCX resistance of PC-3_DCX20 cells estimated with proliferation assay in the presence of DCX (0.25–20 nM). **C** Drug-efflux efficiency in 10 nM DCX-exposed PC-3 WT and PC-3_DCX20 populations, estimated with the calcein efflux assay. **D** Motility (left) and proliferation (middle/right) of PC-3 WT and PC-3_DCX20 cells in the presence of DCX and/or MET estimated with time-lapse videomicroscopy (immediately after 2.5 nM DCX/10 mM MET administration) and Coulter counter (48 h thereafter). **E** Intracellular localization of actin (red), vinculin (green) and  DNA (blue) [left panel] and motility (right panel) of DCX/MET (2.5 nM/10 mM) treated PC-3_DCX20 cells 48 h after DCX/MET administration. **F, G** Apoptotic response of PC-3_DCX20 cells (estimated with annexinV/PI assay, **F**) and their viability [estimated with MTT (**G**, left) and intracellular ATP assays (**G**, right)] after 48-h-long DCX/MET (2.5 nM/10 mM) treatment**.** The statistical significance of the differences was tested for PC-3_DCX20 cells with the Student’s *t*-test (in **B**, **C**, proliferation in **D**, **F**, **G**), or by one-way ANOVA followed by post hoc Tukey’s HSD (motility in **D**, **E**). ^#^*P* ≤ 0.05 versus untreated control; *P ≤ 0.05 as indicated in the charts or 0 mM MET (**D**, middle graph) or PC-3 WT (**F**). All results are representative of at least three independent experiments (*N* ≥ 3). Error bars represent SEM. Note negligible PC-3_DCX20 reactions to the combined DCX/MET treatment

To address the impact of the multi-drug resistance on cellular reactivity to DCX/MET-induced metabolic stress, we employed drug-resistant lineages of PC-3 and DU145 cells. PC-3_DCX20 lineage had been established by a long-term exposition of PC-3 WT cells to increasing doses of DCX [14] (Fig. 2A). Drug resistance of PC-3_DCX20 is illustrated by a considerable mitotic activity in the presence of ≤ 20 nM DCX (Fig. 2B) and drug-efflux efficiency in both the presence and absence of DCX (Fig. 2C, cf. Additional file 1: Fig. S3). A synergy of cytostatic DCX/MET effects in PC-3_DCX20 populations was less pronounced than in their PC-3 WT counterparts. Moderate inhibition of cell motility and proliferation (Fig. 2D, cf. Additional file 1: Fig. S4A) along with negligible changes of PC-3_DCX20 morphology and motility after 48-h-long DCX/MET treatment confirms this notion (Fig. 2E, cf. Additional file 1: Fig. S4B). Concomitantly, it induced considerably less intense apoptotic responses in PC-3_DCX20 than in PC-3 WT populations (Fig. 2F, cf. Additional file 1: Fig. S4C) but had a surprisingly strong effect on NAD(P)H and ATP levels in these cells (Fig. 2G). Adaptative potential of PC-3_DCX20 cells was also confirmed by proteomic studies. They revealed partly overlapping sets of downregulated proteins in both cell lines (i.e., proliferation-related CDKs, SKP1, SMAD2, MAPKs, and STATs). Concomitantly, short-term adaptation responses of PC-3_DCX20 cells encompassed an upregulation of the array of stress- and trafficking-related proteins (CAT, VDACs, CD44, RABs, and TMEDs; Additional file 2: Appendix 1). Corresponding resistance to DCX/MET-induced cytostatic effect and to the metabolic decoupling was observed in drug-resistant DU145_DCX20 populations (Additional file 1: Fig. S5). Even though it was less pronounced than in the case of their PC-3_DCX20 counterparts, our data suggest that acquired drug resistance enhances the efficiency of cell adaptation to chemotherapeutic/metabolic stress.

### Combined DCX/MET stress induces metabolic imbalance in prostate cancer cells

**Fig. 3:**
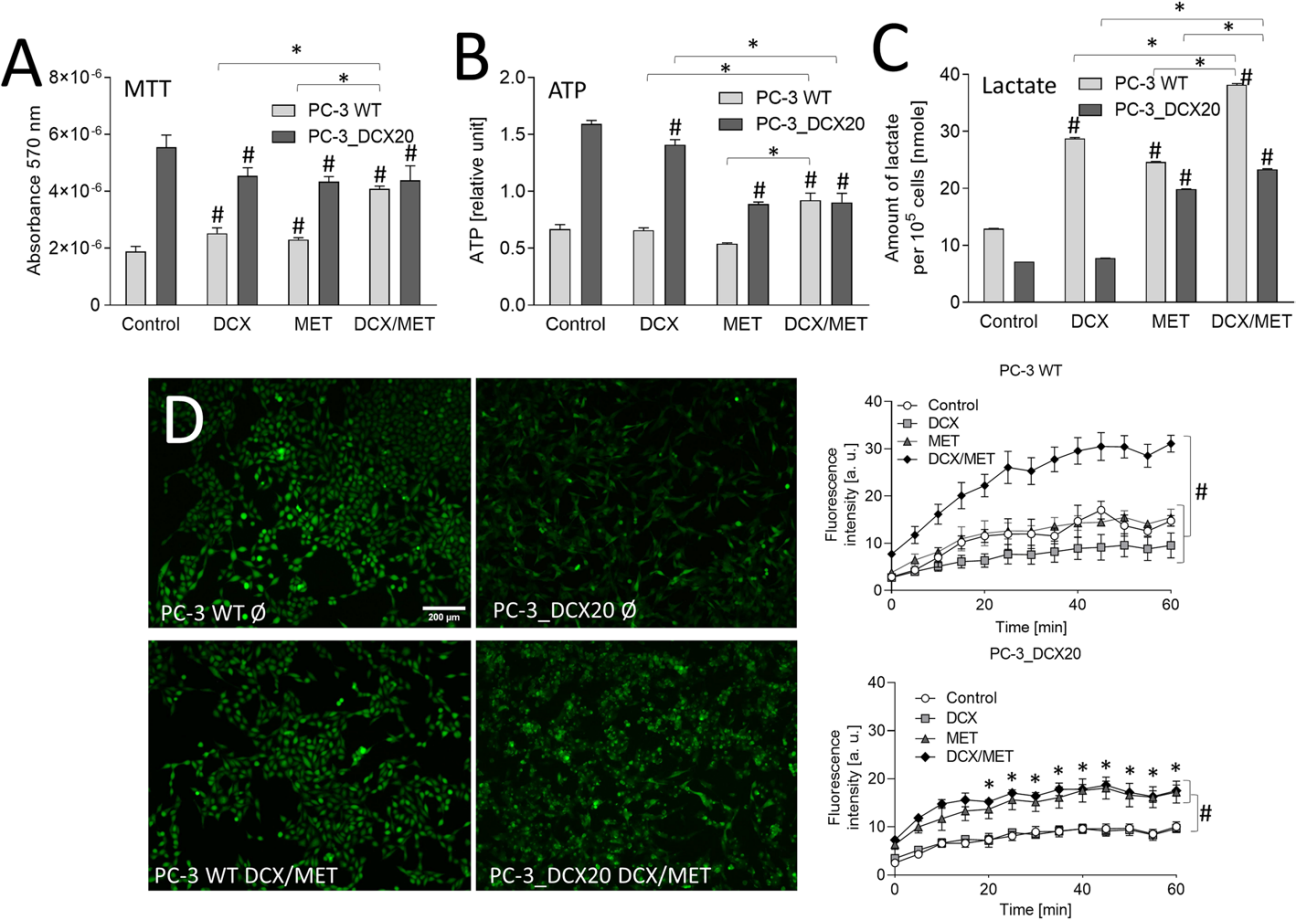
Combined DCX/MET treatment induces metabolic decoupling in PC-3 WT prostate cancer cells. **A**–**C** PC-3 WT and PC-3_DCX20 cells were subjected to DCX and/or MET (2.5 nM/10 mM; 48 h). Metabolic activity (MTT) (**A**), ATP content (**B**), and lactate production (**C**) were analyzed and calculated per 10^5^ cells. **D** Cells were incubated as in **A**, and drug efflux efficiency was measured with a calcein efflux assay. The statistical significance of the differences was tested with Student’s *t*-test (**A**–**D**). ^#^*P* ≤ 0.05 versus untreated control; *P ≤ 0.05 as indicated in the charts or PC-3 WT in **D**. All results are representative of at least three independent experiments (*N* ≥ 3). Error bars represent SEM. Note relatively low MTT/ATP levels and negligible impairment of calcein efflux (metabolic decoupling) in PC-3_DCX20 cells

Further experiments were designed to trace the metabolic status of the cells that managed to survive the long-term (48 h) DCX/MET stress. For this purpose, we normalized intracellular NAD(P)H (MTT assay; Fig. 3A; cf. Fig. 1F) and ATP levels (ATP detection assay; Fig. 3B) against cell numbers in DCX- and/or MET-treated PC-3_DCX20 populations. When administered alone, both agents slightly affected NAD(P)H/ATP levels in PC-3 WT cells. Considerably more increased NAD(P)H/ATP levels were detected in PC-3 WT cells subjected to the combined DCX/MET treatment. In turn, the lack of NAD(P)H accumulation and reduced intracellular ATP levels could be seen in DCX- and/or MET-treated PC-3_DCX20 cells (Fig. 3A, B). This effect was accompanied by the induction of lactate production in MET-treated PC-3_DCX20 cells, showing a compensatory induction of Warburg effect in response to the stress (Fig. 3C). The analyses of MET interference with the drug-efflux efficiency revealed its less pronounced inhibitory effect in drug-resistant PC-3_DCX20 cells (Fig. 3D, cf. Additional file 1: Fig. S3). In conjunction with the less pronounced DCX/MET-evoked growth arrest and apoptosis of PC-3_DCX20 cells (Fig. 2D, F), these data suggest that the acquired drug resistance counteracts the imbalance between NAD(P)H-related energy production and consumption, and alleviates the metabolic overload of prostate cancer cells. Significance of this correlation and its lineage specificity was further confirmed by NAD(P)H/ATP accumulation observed in drug-resistant DU145_DCX20 populations under DCX/MET stress. It correlated with the lack of Warburg effect (i.e., lactate induction), accompanied by a more pronounced impairment of the welfare and drug resistance of DU145_DCX20 than in PC-3_DCX20 cells (cf. Additional file 1: Fig. S6).

### Combined DCX/MET treatment differentially affects ATP production in PC-3 WT and PC-3_DCX20 cells

**Fig. 4:**
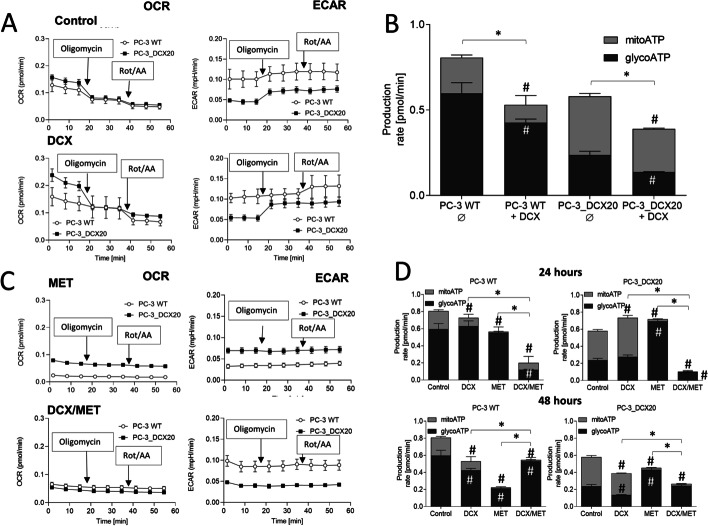
MET induces Warburg effect in prostate cancer cells. **A** OCR/ECAR of control and DCX-treated (48 h) PC-3 WT cells and their PC-3_DCX20 counterparts were analyzed with the Seahorse Analyzer XF HS Mini and XFp software, using the Real-Time ATP Rate Assay [arrows show the timepoints of oligomycin (1 μM) and rotenone/antimycin A application (1 μM/0.5 μM, respectively)]. **B** DCX-induced metabolic microevolution of PC-3 cells visualized by the differences in the metabolic profile (mitoATP versus glycoATP) of PC-3 WT and PC-3_DCX20 cells in the presence/absence of 2.5 nM DCX. **C** OCR/ECAR of MET or DCX/MET-treated (48 h) PC-3 WT and PC-3_DCX20 cells analyzed as in **A**. **D** The effect of MET on the metabolic profile of PC-3 WT and PC-3_DCX20 cells cultivated in the presence/absence of DCX. All the values are calculated per 10^5^ cells. The statistical significance of the differences was tested with Student’s *t*-test; ^#^*P* ≤ 0.05 versus a relevant control; *P ≤ 0.05 versus indicated values counted as the sum of mitoATP and glycoATP. All results are representative of at least three independent experiments (*N* ≥ 3). Error bars represent SEM. Note the inhibition of OXPHOS in DCX/MET-treated PC-3_DCX20 cells

Interrelations between the acquired drug resistance and metabolic coupling of drug-efflux system(s) in prostate cancer cells subjected to chemotherapeutic/metabolic stress prompted us to address DCX and/or MET-induced PC-3 metabolic microevolution. First, Seahorse analyses of PC-3 WT and PC-3_DCX20 metabolic profile enabled us to the assess the events that accompany DCX stress-induced metabolic microevolution of PC-3 populations. Relatively low OCR and high ECAR values estimated for untreated PC-3 WT cells indicate that ATP production in these cells was predominantly based on anaerobic processes (Fig. 4B, cf. A). Short-term DCX treatment further impaired OXPHOS in these cells. In turn, PC-3_DCX20 cells displayed considerably higher OXPHOS rate in control conditions. Because PC-3_DCX20 lineage was established from the long-term DCX-treated PC-3 WT cells, this observation indicates that permanent DCX stress petrifies its OXPHOS phenotype. Differences in the lactate production between PC-3 WT and PC-3_DCX20 cells (cf. Fig. 3C) along with considerably higher NAD(P)H and ATP levels in PC-3_DCX20 cells (cf. Fig. 3A, B) confirm this notion. OXPHOS addiction of PC-3_DCX20 populations was also substantiated by relatively high OXPHOS intensity in the presence of DCX (Fig. 4B; cf. A). In turn, the combined DCX/MET treatment significantly impaired OXPHOS in PC-3_DCX20 cells (Fig. 4D, cf. C). This effect was correlated with the increased ECAR in MET-treated PC-3_DCX20 samples. Finally, a reduction of the total respiration rate (sum of OCR and ECAR) was observed in DCX/MET-treated PC-3_DCX20 cells, but not in their WT counterparts (Fig. 4D). This correlated with the better welfare of PC-3_DCX20 cells and their less pronounced metabolic overload (cf. Figs. 2 and 3). These data indicate that a fine-tuning of ATP production and OXPHOS efficiency determines the metabolic balance and PC-3_DCX20 cell adaptation to the combined chemotherapeutic/metabolic stress.

### Fenofibrate-induced metabolic overload correlates with the high OXPHOS intensity and impaired PC-3 welfare

**Fig. 5:**
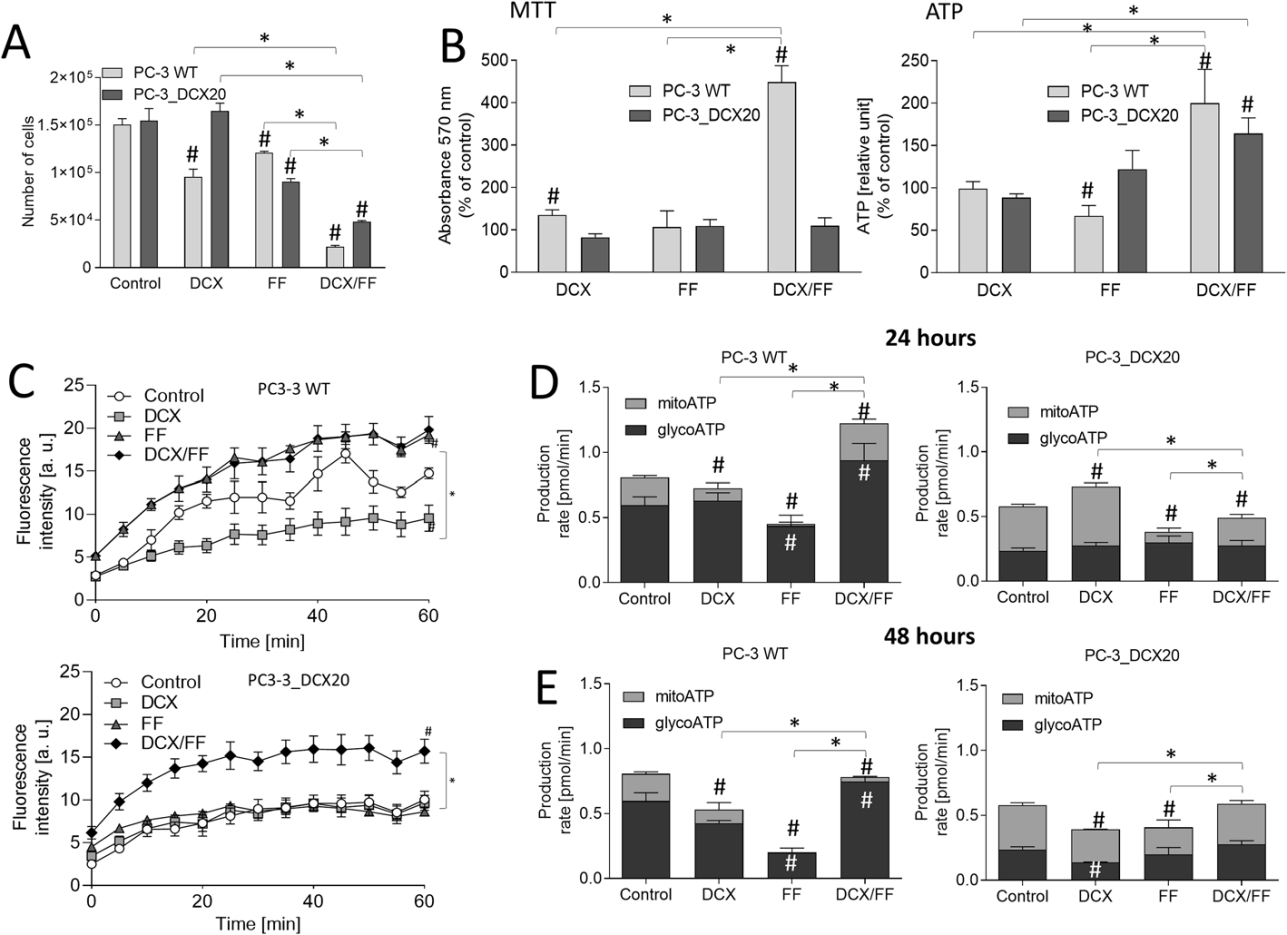
Fenofibrate induces metabolic stress in PC-3_DCX20 cells in the absence of Warburg effect. **A** Proliferation of DCX/FF-treated PC-3 WT and PC-3_DCX20 cells calculated with Coulter counter. **B** NAD(P)H (left) and ATP contents (right) in DCX/FF-treated PC-3 WT and PC-3_DCX20 cells (calculated per 10^5^ cells as percentage of control). **C** Drug-efflux efficiency in DCX/FF-treated PC-3 WT and PC-3_DCX20 cells estimated with calcein efflux assay **D**, **E** Metabolic profile of DCX/FF-treated PC-3 WT and PC-3_DCX20 estimated by Seahorse analyzer after 24 h (**D**) and 48 h (**E**). The statistical significance of the differences was tested with Student’s *t*-test; ^#^*P* ≤ 0.05 versus untreated control (of particular respiration mode in **D** and **E**); *P ≤ 0.05 as indicated in the charts or versus indicated values counted as the sum of mitoATP and glycoATP in **D** and **E**. All results are representative of at least three independent experiments (*N* ≥ 3). Error bars represent SEM. Note the higher OXPHOS intensity and ATP/MDR levels in DCX/FF-treated PC-3_DCX20 cells

To further address the mechanisms underlying cellular adaptation to metabolic stress, we evaluated the synergy of DCX and fenofibrate (FF) effects on the welfare and metabolic profile of PC-3 cells. FF is a PPARα agonist that activates β-oxidation and interferes with the mitochondrial complex I, thus disturbing mitochondrial homeostasis [13]. Again, we observed a downregulation of cell cycle regulators in DCX/FF-treated PC-3 WT and PC-3_DCX20 cells in comparison with DCX-treated cells, accompanied by the upregulation of the factors involved in stress responses and intracellular trafficking (Additional file 3: Appendix 2). However, considerable qualitative and quantitative differences could be seen between MET- and FF-induced responses of DCX-treated PC-3 WT and PC-3_DCX20 cells (cf. Additional file 2: Appendix 1), which confirm their specificity. Relatively strong cytostatic effects of the combined DCX/FF treatment were illustrated by the inhibition of PC-3 WT and PC-3_DCX20 proliferation in the presence of both agents (Fig. 5A). FF exerted stronger cytostatic effects than MET in both PC-3 and DU145 populations (Additional file 1: Fig. S7A). A relatively strong metabolic overload (ATP accumulation) was evident in both DCX/FF-treated PC-3 (Fig. 5B) and DU145 populations (Additional file 1: Fig. S7B, C). This was accompanied by a strongly impaired drug-efflux activity in scarce PC-3_DCX20 (Fig. 5C) and DU145_DCX20 cells that had survived the combined DCX/FF treatment (Additional file 1: Fig. S7D). Finally, PC-3 WT and PC-3_DCX20 cells showed a less pronounced Warburg effect and relatively high total respiration intensity in the presence of DCX/FF (Fig. 5D, E, cf. Additional file 1: Fig. S8). These data confirm that the accumulation of energy carriers in prostate cancer cells impairs their welfare. FF-impaired Warburg effect participates in the less efficient adaptation of drug-resistant cells to the chemotherapeutic/metabolic stress.

### Combined DCX/MET treatment induces EMT in drug-resistant PC-3 cells

**Fig. 6:**
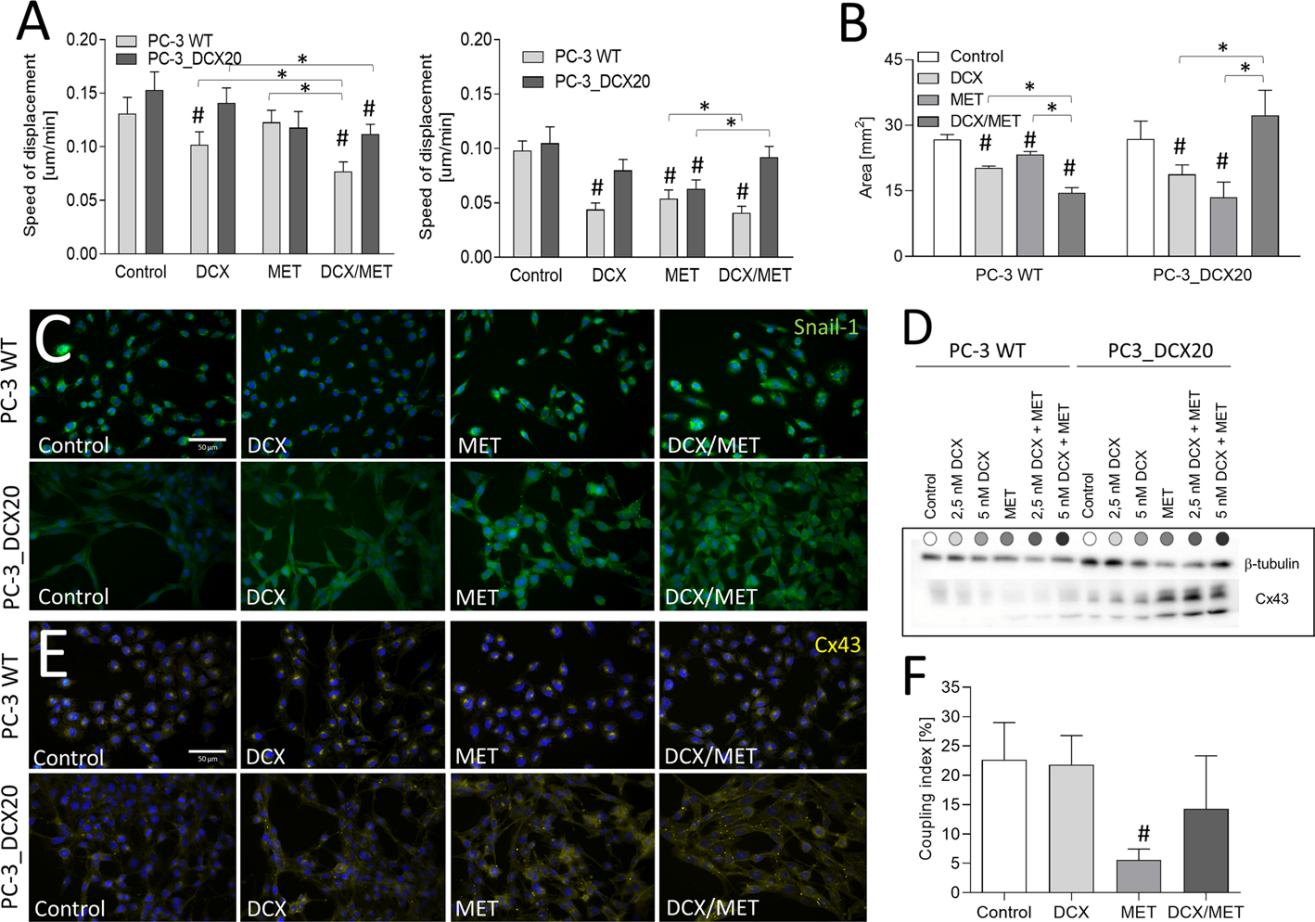
Combined DCX/MET treatment enhances invasive potential of DCX-resistant PC-3 cells. **A** Displacement of PC-3 WT and DCX-resistant PC-3_DCX20 cells was estimated with time-lapse videomicroscopy immediately after the administration of DCX (2.5 nM) and/or MET (10 mM; cf. Additional file 1: Figs. S1 and S4, left panel) or after 48-h-long treatment (right panel). **B** Invasiveness of DCX and/or MET treated cells estimated with Transwell migration assay. **C** Snail-1 expression and localization in PC-3 WT/PC-3_DCX20 cells cultivated in the presence of (2.5 nM) and/or MET (10 mM) [Snail-1 (green), DNA (blue)]. **D**, **E** Cx43 levels and intracellular localization in DCX- and/or MET-treated cells estimated with immunoblotting (**D**, β-tubulin was used as a reference protein) and immunofluorescence (**E**; Cx43: yellow, DNA: blue). **F** GJIC estimated with calcein transfer assay in the absence/presence of AGA (α-glycyrrhetinic acid; 25 μM). Coupling index/ratio represents the percentage of coupled donor cells. The statistical significance of the differences was tested with Student’s *t*-test; #*P* ≤ 0.05 versus untreated control; *P ≤ 0.05 as indicated in the charts. All results are representative of at least three independent experiments (*N* ≥ 3). Error bars represent SEM. Note increased invasiveness of Snail-1/Cx43^high^ cells after DCX/MET

Finally, we focused on the consequences of MET-induced metabolic overload for the invasive phenotype of prostate cancer cells. The upregulation of some EMT-related factors was revealed by mass spectrometry analyses in both PC-3 WT and PC-3_DCX20 populations; however, only in drug-resistant cells was this process translated into pro-invasive phenotypic shifts. This is illustrated by their relatively high displacement capacity in the presence of DCX/MET. After 48-h-long treatment, it reached values similar to those in control conditions and much higher than those seen in DCX/MET-treated PC-3 WT populations (Fig. 6A, cf. Additional file 1: Figs. S1 and S4). Also the induction of cell transmigration potential could be observed in PC-3_DCX20 populations (Fig. 6B). This effect correlated with the upregulation of Snail-1, which is a master EMT regulator in cancer cells (Fig. 6C). Previously unexplored links between metabolic adaptation and invasive phenotype of prostate cancer cells were further confirmed by Cx43 upregulation [36] in PC-3_DCX20 cells exposed to DCX/MET stress (Fig. 6D). This was accompanied by a predominant accumulation of Cx43 in the cytoplasmic compartments of DCX/MET-treated cells with a less evident Cx43 localization in cell-to-cell contacts (Fig. 6E). We did not observe any enhancement of the gap junctional intercellular communication (GJIC) in DCX/MET-treated PC-3_DCX20 cells (Fig. 6F). However, these observations point to Snail-1/Cx43-related EMT, as the process that accompanies the adaptation of drug-resistant cells to the combined chemotherapeutic/metabolic stress.

## Discussion

The application of metabolic blockers has recently been suggested as a means to overcome cancer drug resistance during chemotherapy. This strategy may reduce the effective doses of chemotherapeutic drugs, attenuate their adverse effects, and improve the welfare of the patients subjected to chemotherapy. Owing to the functional links between the drug resistance and invasive (malignant) phenotype of cancer cells [25], metabolic blockers can also affect the progression of the tumors under chemotherapeutic stress. However, the metabolic adaptation of cancer cells is a key obstacle to this approach. It can prompt temporal survival of cancer cells under combined chemotherapeutic/metabolic stress, winning them the time to switch on the adaptative responses [23]. Here, we demonstrate that (1) the metabolic imbalance/decoupling of drug-resistance systems, rather than the energy deficit, accounts for the synergy of cytostatic DCX/MET effects in the populations of drug-sensitive prostate cancer cells. In turn, (2) the high efficiency of drug-efflux systems and the metabolic elasticity of drug-resistant lineages facilitates their survival under the combined DCX/MET-induced stress. Consequently, (3) the acquired drug resistance counteracts the therapeutic effects of metformin, prompting the microevolution of invasive subpopulations.

The activity of drug-resistance systems in prostate cancer cells impairs the efficiency of chemotherapy [1]. Metabolic blockers are commonly believed to interfere with the performance of these systems via impairment of the energy supply for their activity. Actually, we observed an enhancement of DCX sensitivity and inhibition of drug efflux in PC-3 WT and DU145 WT cells upon the combined application of DCX/MET. This finding reveals the inhibitory MET effect on the residual drug resistance of prostate cancer cells. A concomitant metabolic shift (including OXPHOS inhibition) in DCX/MET-treated cells confirmed the relationships between drug-sensitizing MET effects and the welfare of the mitochondria [17, 37, 38]. Activation of glycolysis may partly compensate for this effect, whereas the block of OXPHOS and intensified PARP-dependent repair of DCX/MET-induced DNA aberrations [39] collectively account for the high NAD(P)H levels in DCX/MET-treated PC-3 WT and DU145 WT cells. However, concomitant ATP accumulation suggests the metabolic decoupling in these cells, rather than their energy deficit. Along with the handicapped drug-efflux efficiency, these results demonstrate the impairment of transmission routes between bioenergetic machinery and drug-resistance systems in DCX/MET-treated cells. It remains to be elucidated whether ATP and NAD(P)H accumulation in DCX/MET-treated may partly compensate for the DCX-induced stress. However, the metabolic imbalance primarily accounts for the synergy of cytotoxic DCX/MET effects in prostate cancer cell populations.

Studies on the drug-resistant PC-3/DU145 lineages helped us to identify the systems that control metabolic coupling in DCX/MET-treated cancer cells. The signs of the synergy of DCX and MET effects in PC-3_DCX20 and DU145_DCX20 populations partly confirmed the potential of MET for the palliative treatment of drug-resistant prostate tumors. However, relatively mild cytostatic/pro-apoptotic responses of these cells to the combined DCX/MET-induced stress indicate that efficient drug-efflux systems limit the harmful effects of DCX on metabolic homeostasis. Additionally, the short- and long-term adaptation responses of these cells to bioenergetic challenges can be enhanced by their stress-induced microevolution toward metabolically elastic phenotype [24]. This notion is supported by the signs of Warburg effect observed in PC-3 WT populations during their long-term DCX-induced microevolution toward chemoresistant phenotype. Maintenance of high NAD(P)H/ATP levels in control PC-3_DCX20 cells can additionally facilitate their adaptation to DCX/MET-induced stress by securing energy reserves for a prompt efflux activation. This system is further complemented by the metabolic elasticity of PC-3_DCX20 cells, illustrated by DCX/MET-induced shifts from aerobic to anaerobic metabolic profile and their “metabolic idleness.” Along with the relatively high viability of DCX/MET treated PC-3_DCX20 cells, these data demonstrate that a fine-tuning of metabolic profile and activity [26] participates in PC-3_DCX20 adaptability to the metabolic stress. It secures the metabolic coupling of P-gp-related drug efflux systems, which are active in the analyzed cell lines [10]. Relatively high OXPHOS intensity, NAD(P)H levels, and ATP production were accompanied by strongly impaired welfare of DCX/FF-treated cells. FF activates lipid β-oxidation in a PPARα-dependent manner [13, 40] and interferes with the function of mitochondrial complex I [41]. Consequently, it can impair a delicate balance between mitochondrial energy supply and demand, compromising the cell adaptation to the metabolic stress. Thus, the fine-tuning of cellular metabolism supports drug-efflux systems during cell adaptation to metabolic stress by preventing metabolic uncoupling and mitochondrial overload.

Collectively, we demonstrate the crucial role of the cooperation between the acquired drug resistance and metabolic plasticity in the adaptation of prostate cancer cells to the combined chemotherapeutic/metabolic stress. A high activity of drug-efflux systems in drug-resistant cells “wins them the time” to finely tune their metabolic phenotype. A balance of energy supplies (e.g., OXPHOS and glycolysis) and demand (e.g., cell motility, proliferation, drug efflux, and repair systems) preserves the welfare of drug-resistant prostate cancer cells, underlying the strategy of their “metabolic escape.” Slightly higher DCX/MET sensitivity of DU145_DCX20 cells, accompanied by the signs of NAD(P)H/ATP accumulation and a more prominent drug-efflux impairment in these cells (in the absence of Warburg effect), supports this notion. Notably, metformin can also activate autophagy in cancer cells [26]. It can interfere with cancer growth or promote its pro-invasive microevolution in a context-dependent manner [6, 42]. Further studies are necessary to elucidate the potential links between autophagic cell responses and the phenotypic shifts (including AMPK activation, redox signaling, and metabolic reprogramming [43]) in our experimental model. However, a metformin-induced, self-protective autophagy may facilitate metabolic escape of drug-resistant cells.

Alternatively, a “physical escape” strategy of cancer cell adaptation to the DCX/MET-induced stress is indicated by the links between the metabolic and invasive phenotype of cancer cells [44, 45]. They are illustrated by the Snail-1/Cx43 upregulation and EMT induction in DCX/MET-treated drug-resistant cells. Direct involvement of both proteins in EMT of prostate cancer cells has previously been reported [36, 46]. We did not reveal GJIC involvement in the adaptation of PC-3_DCX20 cells to DCX/MET-induced stress (not shown). Neither did DCX/FF-treatment upregulate Cx43 in these cells (not shown), even though it has previously been shown to trigger an expansion of super-resistant and invasive prostate cancer cell lineages from CD44^+^ cells [28]. Our data indicate that Cx43 can cooperate with Snail-1 via the (hemi)channel-(in)dependent control of mitochondrial stress [36, 47, 48] and cell invasiveness [33, 49–51]. However, the changes of their functional status in DCX/MET-treated cells are just “a tip of the iceberg.” This notion is illustrated by proteomic studies, which detected (somewhat expected) downregulation of proliferation-related factors in both PC-3 lineages, accompanied by the shifts in the expression of an array of mutually interlinked stress-related proteins in DCX/MET-treated PC-3_DCX20 cells compared to their DCX-treated counterparts. These include the upregulation of stress-responsive mitochondrial proteins (VDACs), catalase, the regulators of intracellular trafficking (RABs/TMEDs), and CD44, which is a marker of cellular stemness. An open question remains, whether the repeated DCX/MET-treatment cycles can petrify these adaptative shifts, i.e., trigger a full-scale adaptative microevolution of prostate cancer cells. Further studies are also necessary to determine whether these reactions are induced in a “Lamarckian” fashion, or the adaptation is achieved at the population level through the “Darwinian” selective survival/expansion of pre-EMT cells. [52]. However, the interrelations between the drug resistance, metabolic plasticity, and cell invasiveness [27, 53, 54] apparently underlie cellular adaptation to the combined chemotherapeutic/metabolic stress. Additionally, different patterns of proteomic cell responses to DCX/MET and DCX/FF treatment indicate that the quality and quantity of the signal (MET versus FF) can bias the direction of microevolution toward different scenarios.

## Conclusions

Combinations of metabolic blockers with conventional cytostatic drugs provide a way to improve cancer treatment efficiency and reduce their effective doses. This strategy may be beneficial for elderly patients, who are usually more vulnerable to the adverse effects of prostate cancer chemotherapy. Metformin has long been suggested as a systemically neutral antidiabetic agent that may also enhance the efficiency of conventional anticancer approaches and prolong the time of tumor recurrence. Therefore, the optimization of combined diabetes/prostate cancer treatment appears as the fundamental aspect of palliative clinical practice. Our study partly confirmed the suitability of metformin for the combined prostate cancer therapy. It showed that the metabolic decoupling of drug-resistance system(s) rather than energy deficit impairs the acquired drug resistance of prostate cancer cells. However, it also revealed their lineage-specific adaptative responses and the onset of phenotypic microevolution toward the invasive phenotype induced by metformin. Accordingly, the links between the drug resistance, metabolic balance, and invasive phenotype of prostate cancer cells pose limitations for the cytostatic activity of metformin and may explain its low efficiency reported in cohort/metadata analyses [19]. Potential consequences of metformin treatment for elderly patients, who are parallelly subjected to anticancer treatment, justify further efforts to unravel the mechanisms underlying the adaptation responses to the combined chemotherapeutic/metabolic stress.

## Supplementary Information

**Additional file 1. Supplementary data: Figure S1.** Responsiveness of PC-3 WT cell to the combined DCX/MET treatment; **Figure S2.** Metformin increases the sensitivity of DU145 cells to docetaxel; **Figure S3.** Effect of DCX/MET on PC-3 drug-resistance; **Figure S4.** PC-3_DCX20 responsiveness to the combined DCX (2.5 nM)/MET (10 mM) treatment; **Figure S5.** The sensitivity of DU145_DCX20 cells to the combined DCX/MET treatment; **Figure S6.** DCX/MET-induced metabolic decoupling in prostate cancer DU145 populations; **Figure S7.** Effect of combined DCX/FF treatment on the metabolic balance in prostate cancer DU145 cells; **Figure S8.** Metabolic profile of DCX/FF-treated PC-3 WT and PC-3_DCX20 cells.

**Additional file 2. Appendix 1:** Proteomic analyses of DCX/MET-treated PC-3 WT and PC-3_DCX20 cells.

**Additional file 3. Appendix 2:** Proteomic analyses of DCX/FF-treated PC-3 WT and PC-3_DCX20 cells.

## Data Availability

Data related to the manuscript are available from the authors upon a reasonable request.

## Abbreviations

BP: Bandpass
BSA: Bovine serum albumin
Cx43: Connexin 43
DMEM: Dulbecco’s modified Eagle medium
DCX: Docetaxel
EMT: Epithelial–mesenchymal transition
FACS: Fluorescence-activated cell sorter
FF: Fenofibrate
HDL/LDL: High-/low-density lipoprotein
MDR: Multi-drug resistance
MET: Metformin
OXPHOS: Oxidative phosphorylation
PPARα: Peroxisome proliferator-activated receptor-α
ROS: Reactive oxygen species

## References

[1] Bell CC, Gilan O (2020). Principles and mechanisms of non-genetic resistance in cancer. Br J Cancer.

[2] Catalano A, Iacopetta D, Ceramella J, Scumaci D, Giuzio F, Saturnino C (2022). Multidrug resistance (MDR): a widespread phenomenon in pharmacological therapies. Molecules.

[3] Szakács G, Paterson JK, Ludwig JA, Booth-Genthe C, Gottesman MM (2006). Targeting multidrug resistance in cancer. Nat Rev Drug Discov.

[4] Housman G, Byler S, Heerboth S, Lapinska K, Longacre M, Snyder N (2014). Drug resistance in cancer: an overview. Cancers (Basel).

[5] Siegel RL, Miller KD, Fuchs HE, Jemal A (2022). Cancer statistics, 2022. CA Cancer J Clin.

[6] Huang T, Song X, Yang Y, Wan X, Alvarez AA, Sastry N, Feng H, Hu B, Cheng S-Y (2018). Autophagy and hallmarks of cancer. Crit Rev Oncog.

[7] Karatsai O, Stasyk O, Redowicz MJ (2020). Effects of arginine and its deprivation on human glioblastoma physiology and signaling. Adv Exp Med Biol.

[8] Gao X, Sanderson SM, Dai Z, Reid MA, Cooper DE, Lu M, Nichenametla SN, Locasale JW (2019). Dietary methionine influences therapy in mouse cancer models and alters human metabolism. Nature.

[9] Icard P, Loi M, Wu Z, Icard P, Loi M, Wu Z (2021). Metabolic strategies for inhibiting cancer development. Adv Nutr.

[10] Chen CL, Lin CY, Kung HJ (2021). Targeting mitochondrial OXPHOS and their regulatory signals in prostate cancers. Int J Mol Sci.

[11] Ngoi NYL, Eu JQ, Hirpara J, Wang L, Lim JSJ, Lee SC (2020). Targeting cell metabolism as cancer therapy. Antioxid Redox Signal.

[12] Theile D, Wizgall P (2021). Acquired ABC-transporter overexpression in cancer cells: transcriptional induction or Darwinian selection?. Naunyn-Schmiedeb Arch Pharmacol.

[13] Lian X, Wang G, Zhou H, Zheng Z, Fu Y, Cai L (2018). Anticancer properties of fenofibrate: a repurposing use. J Cancer.

[14] Luty M, Piwowarczyk K, Łabędź-Masłowska A, Wróbel T, Szczygieł M, Catapano J (2019). Fenofibrate augments the sensitivity of drug-resistant prostate cancer cells to docetaxel. Cancers (Basel).

[15] Hirpara J, Eu JQ, Tan JKM, Wong AL, Clement MV, Kong LR (2019). Metabolic reprogramming of oncogene-addicted cancer cells to OXPHOS as a mechanism of drug resistance. Redox Biol.

[16] Delma MI (2018). Three may be better than two: a proposal for metformin addition to PI3K/Akt inhibitor-antiandrogen combination in castration-resistant prostate cancer. Cureus.

[17] Ippolito L, Marini A, Cavallini L, Morandi A, Pietrovito L, Pintus G (2016). Metabolic shift toward oxidative phosphorylation in docetaxel resistant prostate cancer cells. Oncotarget.

[18] Samuel SM, Varghese E, Koklesová L, Líšková A, Kubatka P, Büsselberg D (2020). Counteracting chemoresistance with metformin in breast cancers: targeting cancer stem cells. Cancers (Basel).

[19] Feng Z, Zhou X, Liu N, Wang J, Chen X, Xu X (2019). Metformin use and prostate cancer risk: a meta-analysis of cohort studies. Medicine.

[20] Coyle C, Cafferty FH, Vale C, Langley RE (2016). Metformin as an adjuvant treatment for cancer: a systematic review and meta-analysis. Ann Oncol.

[21] Schoonjans CA, Gallez B. Metabolic plasticity of tumor cells: how they do adapt to food deprivation. In: Advances in Experimental Medicine and Biology. 2020;pp. 109–123. 10.1007/978-3-030-34025-4_6 32130696

[22] Bokil A, Sancho P (2019). Mitochondrial determinants of chemoresistance. Cancer Drug Resist.

[23] Hönigova K, Navratil J, Peltanova B, Polanska HH, Raudenska M, Masarik M (2022). Metabolic tricks of cancer cells. Biochim Biophys Acta Rev Cancer.

[24] Andrzejewski S, Siegel PM, St-Pierre J (2018). Metabolic profiles associated with metformin efficacy in cancer. Front Endocrinol.

[25] Shuvalov O, Daks A, Fedorova O, Petukhov A, Barlev N (2021). Linking metabolic reprogramming, plasticity and tumor progression. Cancers (Basel).

[26] Cioce M, Pulito C, Strano S, Blandino G, Fazio VM (2020). Metformin: metabolic rewiring faces tumor heterogeneity. Cells.

[27] Caino MC, Altieri DC (2015). Cancer cells exploit adaptive mitochondrial dynamics to increase tumor cell invasion. Cell Cycle.

[28] Wróbel T, Luty M, Catapano J, Karnas E, Szczygieł M, Piwowarczyk K (2020). CD44^+^ cells determine fenofibrate-induced microevolution of drug-resistance in prostate cancer cell populations. Stem Cells.

[29] Koczurkiewicz P, Podolak I, Wójcik KA, Galanty A, Madeja Z, Michalik M (2013). Lclet 4 enhances pro-apoptotic and anti-invasive effects of mitoxantrone on human prostate cancer cells—in vitro study. Acta Biochim Pol.

[30] Sroka J, Antosik A, Czyż J, Nalvarte I, Olsson JM, Spyrou G (2007). Overexpression of thioredoxin reductase 1 inhibits migration of HEK-293 cells. Biol Cell.

[31] Kochanowski P, Catapano J, Pudełek M, Wróbel T, Madeja Z, Ryszawy D (2021). Temozolomide induces the acquisition of invasive phenotype by O6-methylguanine-DNA methyltransferase (MGMT)^+^ glioblastoma cells in a Snail-1/Cx43-dependent manner. Int J Mol Sci.

[32] Szpak K, Wybieralska E, Niedziałkowska E, Rak M, Bechyne I, Michalik M (2011). DU-145 prostate carcinoma cells that selectively transmigrate narrow obstacles express elevated levels of Cx43. Cell Mol Biol Lett.

[33] Piwowarczyk K, Paw M, Ryszawy D, Rutkowska-Zapała M, Madeja Z, Siedlar M (2017). Connexin43^high^ prostate cancer cells induce endothelial connexin43 up-regulation through the activation of intercellular ERK1/2-dependent signaling axis. Eur J Cell Biol.

[34] Paw M, Borek I, Wnuk D, Ryszawy D, Piwowarczyk K, Kmiotek K (2017). Connexin43 controls the myofibroblastic differentiation of bronchial fibroblasts from patients with asthma. Am J Respir Cell Mol.

[35] Daniel-Wójcik A, Misztal K, Bechyne I, Sroka J, Miekus K, Madeja Z (2008). Cell motility affects the intensity of gap junctional coupling in prostate carcinoma and melanoma cell populations. Int J Oncol.

[36] Ryszawy D, Sarna M, Rak M, Szpak K, Kȩdracka-Krok S, Michalik M (2014). Functional links between Snail-1 and CX43 account for the recruitment of CX43-positive cells into the invasive front of prostate cancer. Carcinogenesis.

[37] Zhang HH, Guo XL (2016). Combinational strategies of metformin and chemotherapy in cancers. Cancer Chemother Pharmacol.

[38] Deng J, Peng M, Wang Z, Zhou S, Xiao D, Deng J (2019). Novel application of metformin combined with targeted drugs on anticancer treatment. Cancer Sci.

[39] Murata MM, Kong X, Moncada E, Chen Y, Imamura H, Wang P (2019). NAD+ consumption by PARP1 in response to DNA damage triggers metabolic shift critical for damaged cell survival. Mol Biol Cell.

[40] Grabacka M, Pierzchalska M, Dean M, Reiss K (2016). Regulation of ketone body metabolism and the role of PPARα. Int J Mol Sci.

[41] Wilk A, Wyczechowska D, Zapata A, Dean M, Mullinax J, Marrero L (2015). Molecular mechanisms of fenofibrate-induced metabolic catastrophe and glioblastoma cell death. Mol Cell Biol.

[42] White E (2015). The role for autophagy in cancer. J Clin Invest.

[43] Gwangwa MV, Joubert AM, Visagie MH (2018). Crosstalk between the Warburg effect, redox regulation and autophagy induction in tumourigenesis. Cell Mol Biol Lett.

[44] Srihari S, Kwong R, Tran K, Simpson R, Tattam P, Smith E (2018). Metabolic deregulation in prostate cancer. Mol Omics.

[45] Montanari M, Rossetti S, Cavaliere C, D’Aniello C, Malzone MG, Vanacore D (2017). Epithelial–mesenchymal transition in prostate cancer: an overview. Oncotarget.

[46] Smith BN, Odero-Marah VA (2012). The role of Snail in prostate cancer. Cell Adh Migr.

[47] Shimura D, Nuebel E, Baum R, Valdez SE, Xiao S, Warren JS (2021). Protective mitochondrial fission induced by stress-responsive protein GJA1-20k. eLife.

[48] Gielen PR, Aftab Q, Ma N, Chen VC, Hong X, Lozinsky S (2013). Connexin43 confers temozolomide resistance in human glioma cells by modulating the mitochondrial apoptosis pathway. Neuropharmacology.

[49] Piwowarczyk K, Kwiecień E, Sośniak J, Zimoląg E, Guzik E, Sroka J (2018). Fenofibrate interferes with the diapedesis of lung adenocarcinoma cells through the interference with Cx43/EGF-dependent intercellular signaling. Cancers (Basel).

[50] Piwowarczyk K, Wybieralska E, Baran J, Borowczyk J, Rybak P, Kosińska M (2015). Fenofibrate enhances barrier function of endothelial continuum within the metastatic niche of prostate cancer cells. Expert Opin Ther Targets.

[51] Czyż J (2008). The stage-specific function of gap junctions during tumourigenesis. Cell Mol Biol Lett.

[52] Easwaran H, Tsai HC, Baylin SB (2014). Cancer epigenetics: tumor heterogeneity, plasticity of stem-like states, and drug resistance. Mol Cell.

[53] Bakir B, Chiarella AM, Pitarresi JR, Rustgi AK, Rustgi AK (2020). EMT, MET, plasticity, and tumor metastasis. Trends Cell Biol.

[54] D’Alterio C, Scala S, Sozzi G, Roz L, Bertolini G (2020). Paradoxical effects of chemotherapy on tumor relapse and metastasis promotion. Semin Cancer Biol.

