# Supplementary material for: Acquired drug resistance interferes with the susceptibility of prostate cancer cells to metabolic stress
Source: Cell Mol Biol Lett. 2022 Nov 18;27:100. doi: 10.1186/s11658-022-00400-1 (PMC9673456; doi:10.1186/s11658-022-00400-1)
Supplement: Supplementary file 1 — Additional file 1. Supplementary data: Figure S1. Responsiveness of PC-3 WT cell to the combined DCX/MET treatment; Figure S2. Metformin increases the sensitivity of DU145 cells to docetaxel; Figure S3. Effect of DCX/MET on PC-3 drug-resistance; Figure S4. PC-3_DCX20 responsiveness to the combined DCX (2.5 nM)/MET (10 mM) treatment; Figure S5. The sensitivity of DU145_DCX20 cells to the combined DCX/MET treatment; Figure S6. DCX/MET-induced metabolic decoupling in prostate cancer DU145 populations; Figure S7. Effect of combined DCX/FF treatment on the metabolic balance in prostate cancer DU145 cells; Figure S8. Metabolic profile of DCX/FF-treated PC-3 WT and PC-3_DCX20 cells. [file 11658_2022_400_MOESM1_ESM.docx]

**SUPPLEMENTARY MATERIAL**

**Acquired drug-resistance interferes with the susceptibility of prostate cancer cells to metabolic stress**

Jessica Catapano^1^, Marcin Luty^1^, Tomasz Wróbel^1^, Maciej Pudełek^1^, Katarzyna Piwowarczyk^1^, Sylwia Kędracka-Krok^2^, Maciej Siedlar^3^, Zbigniew Madeja^1^, Jarosław Czyż^1^

^1^Department of Cell Biology, Faculty of Biochemistry, Biophysics and Biotechnology, Jagiellonian University in Kraków, ul. Gronostajowa 7, 30-387 Kraków, Poland;

^2^Department of Physical Biochemistry, Faculty of Biochemistry, Biophysics and Biotechnology, Jagiellonian University, Gronostajowa 7, 30-387 Kraków; and Proteomics and Mass Spectrometry Laboratory, Malopolska Centre of Biotechnology, Jagiellonian University, Gronostajowa 7A, 30-387 Kraków, Poland

^3^Department of Clinical Immunology, Institute of Pediatrics, Faculty of Medicine, Jagiellonian University Medical College, Wielicka 265, 30-663 Kraków, Poland


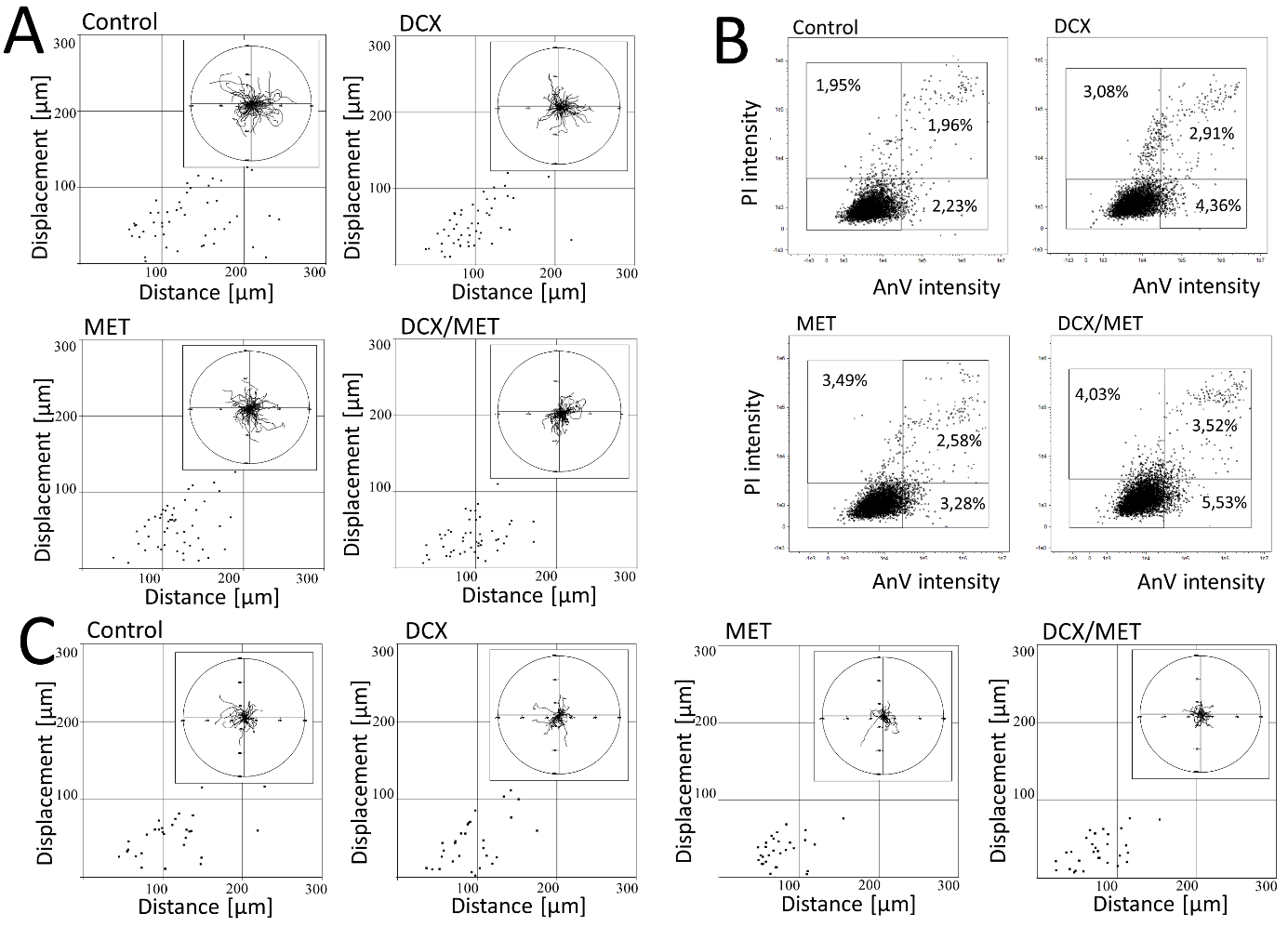


**Figure S1. Responsiveness of PC-3 WT cell to the combined DCX/MET treatment.** **(A)** Trajectories and movement parameters of single PC-3 WT cells estimated with time-lapse videomicroscopy immediately after DCX (2.5 nM) and/or MET (10 mM) administration. Cell trajectories are depicted as circular diagrams (axis scale in µm) drawn with the initial point of each trajectory placed at the origin of the plot (registered for 8 hours; N > 50). Dot-plots show movement parameters of single cells. **(B)** Dot-plots showing the apoptotic response of PC-3 WT cells to the long-term DCX/MET treatment (48 hours; 2.5 nM/10 mM) estimated with AnnexinV/PI assay. Compensated dot-plots comprise 50 000 events, classified based on their bright field ratio. **(C)** Trajectories and movement parameters of single PC-3 WT cells estimated 48 hours after DCX (2.5 nM) and/or MET (10 mM) administration. **Note the additive effects of DCX/MET on the viability of PC-3 WT cells.**

**
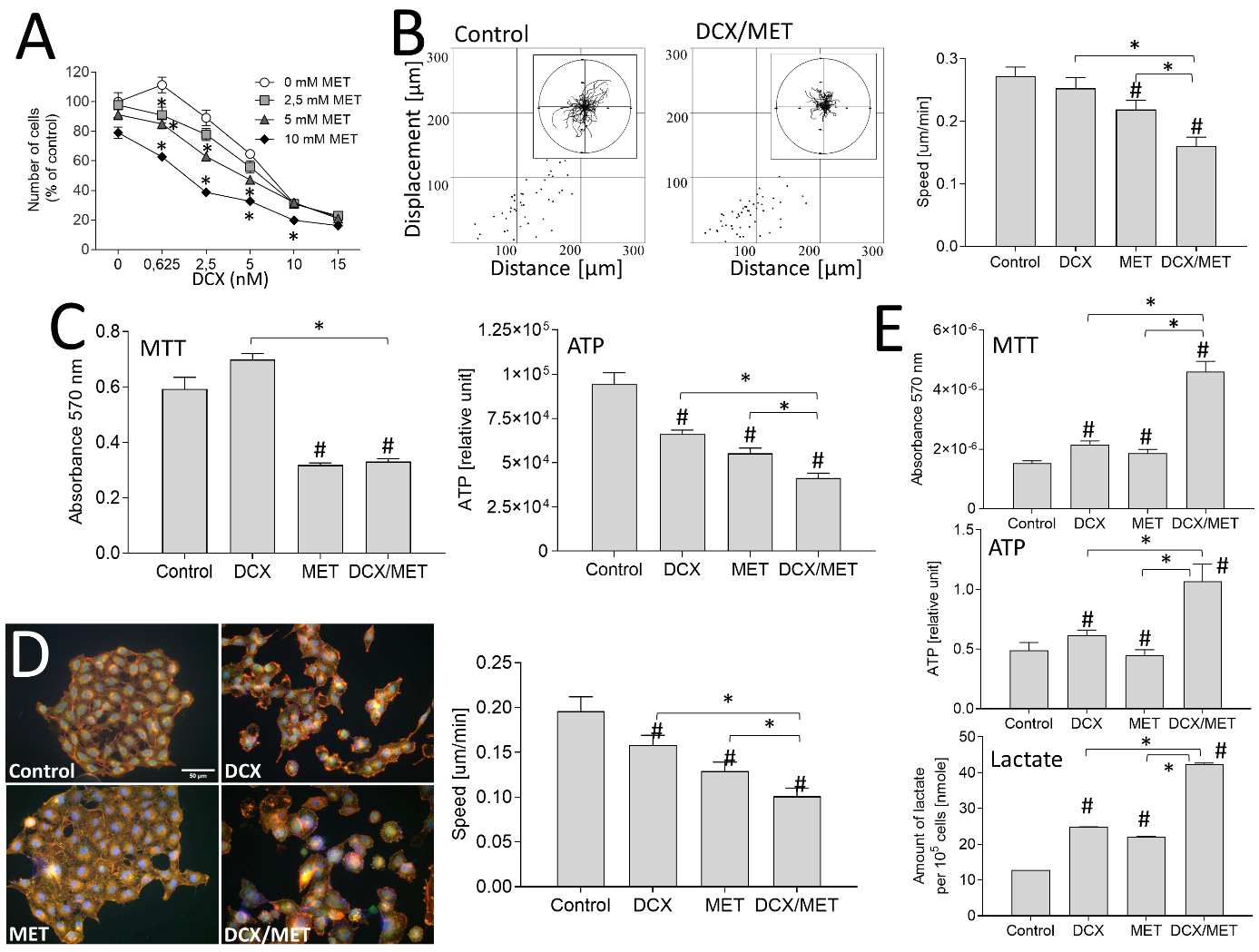
**

**Figure S2. Metformin increases the sensitivity of DU145 cells to docetaxel. (A)** Effect of DCX (0,625-15 nM) and/or MET (2.5-10 mM) on DU145 proliferation **(B)** Motility of DU145 cells estimated directly after DCX (2.5 nM) and/or MET (10 mM) administration with time-lapse videomicroscopy. **(C)** Effect of the long-term (48 hours) DCX/MET (2.5 nM/10 mM) treatment on the viability of DU145 cell populations, estimated with MTT assay and ATP detection kit. **(D)** Actin cytoskeleton architecture (F-actin: red; vinculin: green; DNA: blue) and motility estimated in long-term (48 hours) DCX/MET (2,5 nM/10 mM) treated DU145 cells. **(E)** Metabolic profile of DU145 cells after the long-term (48 hours) DCX/MET (2,5 nM/10 mM) treatment estimated with MTT, ATP and lactate assay. The data are calculated per 10^5^ cells. The statistical significance of the differences was tested with t-Student test (in A, C, E), or by one-way ANOVA followed by post hoc Tukey's HSD (in B, D). #P ≤ 0.05 vs untreated control; *P ≤ 0.05 as indicated in the charts or 0 mM MET in A. All results are representative of a least three independent experiments (N ≥ 3). Error bars represent SEM. **Note the similar DCX/MET reactivity of DU145 and PC-3 cells (cf. Fig. 1).**

**
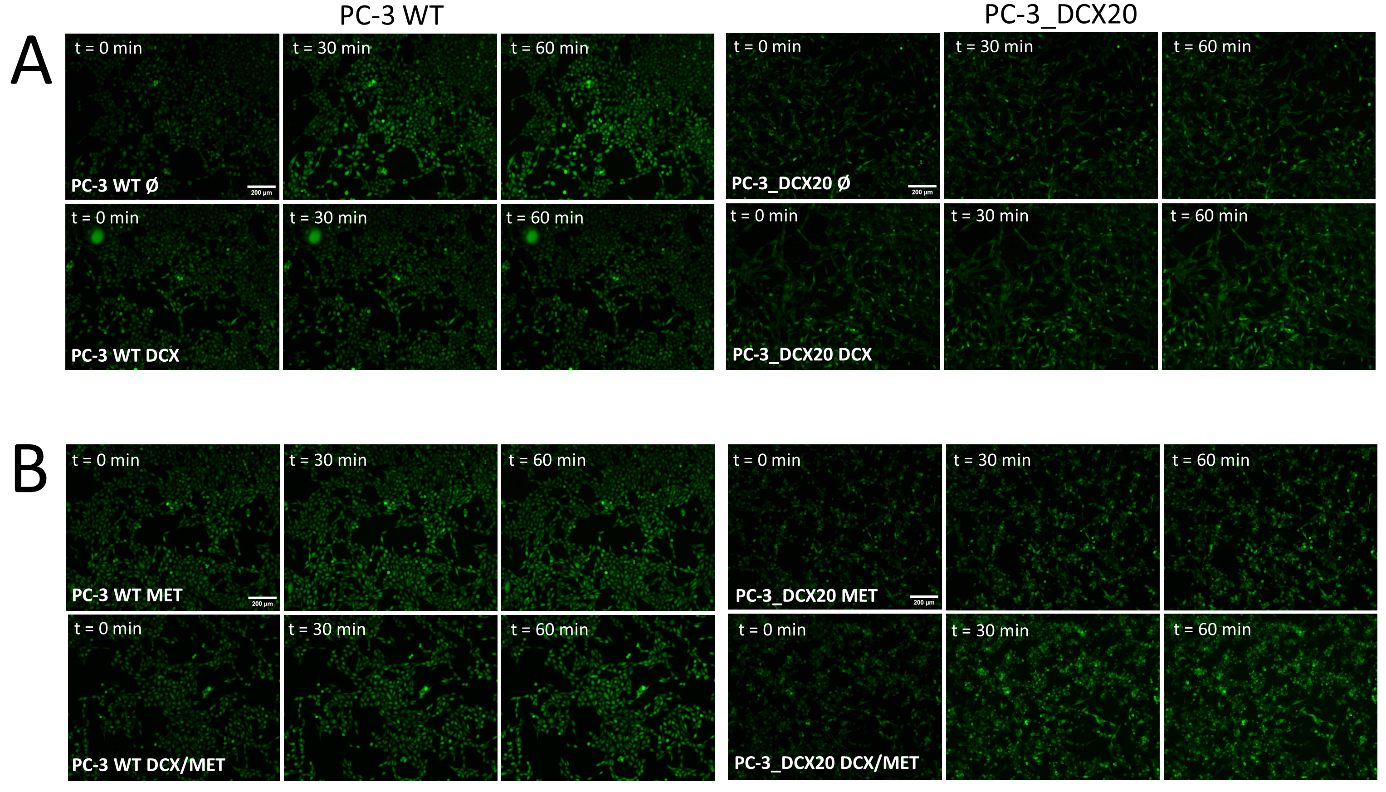
**

**Figure S3. Effect of DCX/MET on PC-3 drug-resistance.** Representative images of PC-3 WT and PC-3_DCX20 populations cultivated in the presence of calcein AM for 30 and 60 min. Scale bar = 200 µm. **Note the enhanced calcein accumulation in the cells subjected to the combined DCX/MET treatment**.

***
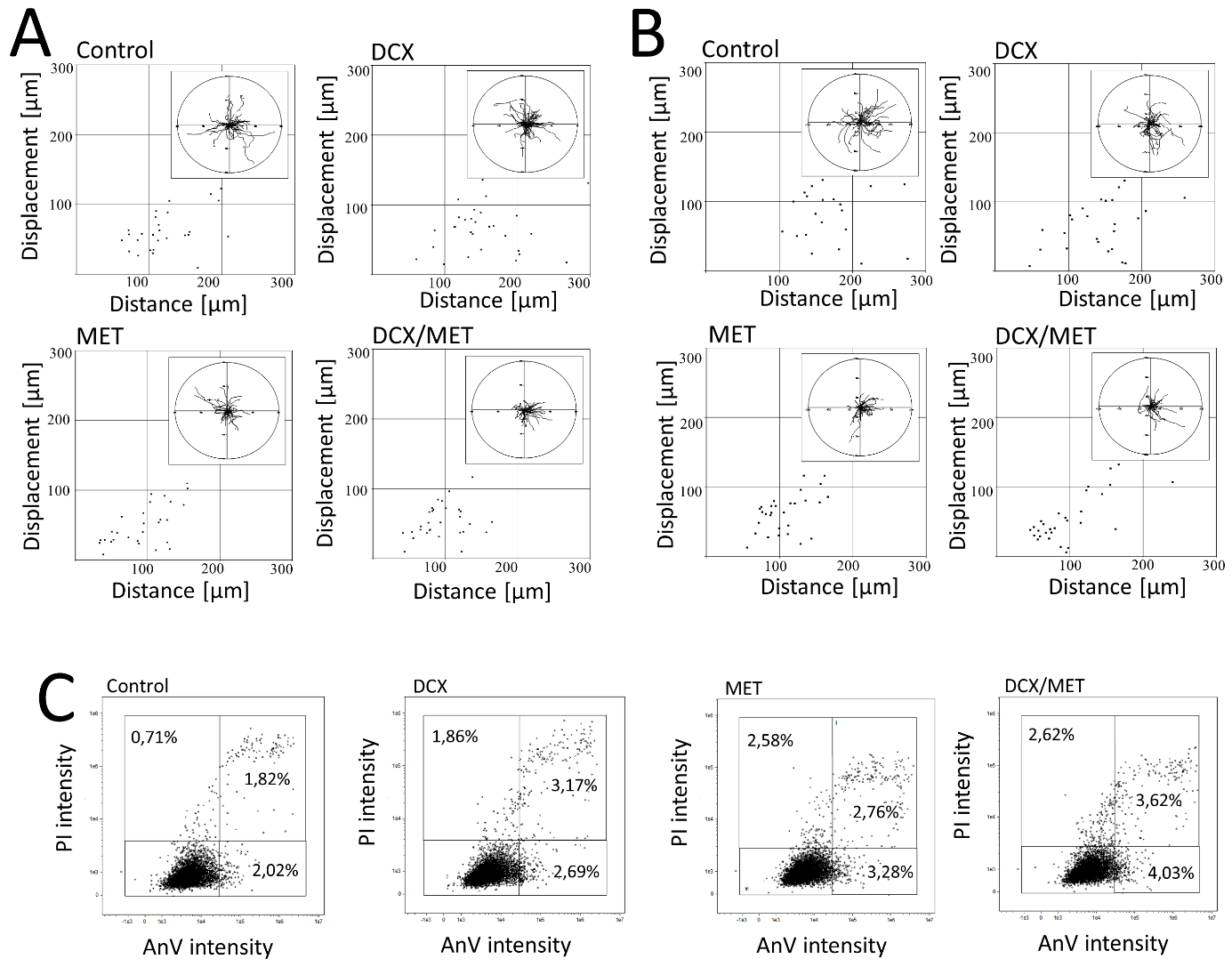
***

**Figure S4. PC-3_DCX20 responsiveness to the combined DCX (2.5 nM)/MET (10 mM) treatment. (A)** Trajectories and movement parameters of single PC-3_DCX20 cells estimated immediately after DCX (2.5 nM) and/or MET (10 mM) administration with time-lapse videomicroscopy. **(B)** Trajectories and movement parameters of single PC-3_DCX20 cells estimated 48 hours after DCX (2.5 nM) and/or MET (10 mM) administration. **(C)** Compensated dot-plots showing the apoptotic response of PC-3_DCX20 cells to the long-term DCX/MET treatment (2.5 nM/10 mM) estimated with AnnexinV/PI assay (50 000 events, classified based on their bright field ratio). **Note the relatively negligible cytostatic effects of DCX/MET in PC-3_DCX20 populations.**

***
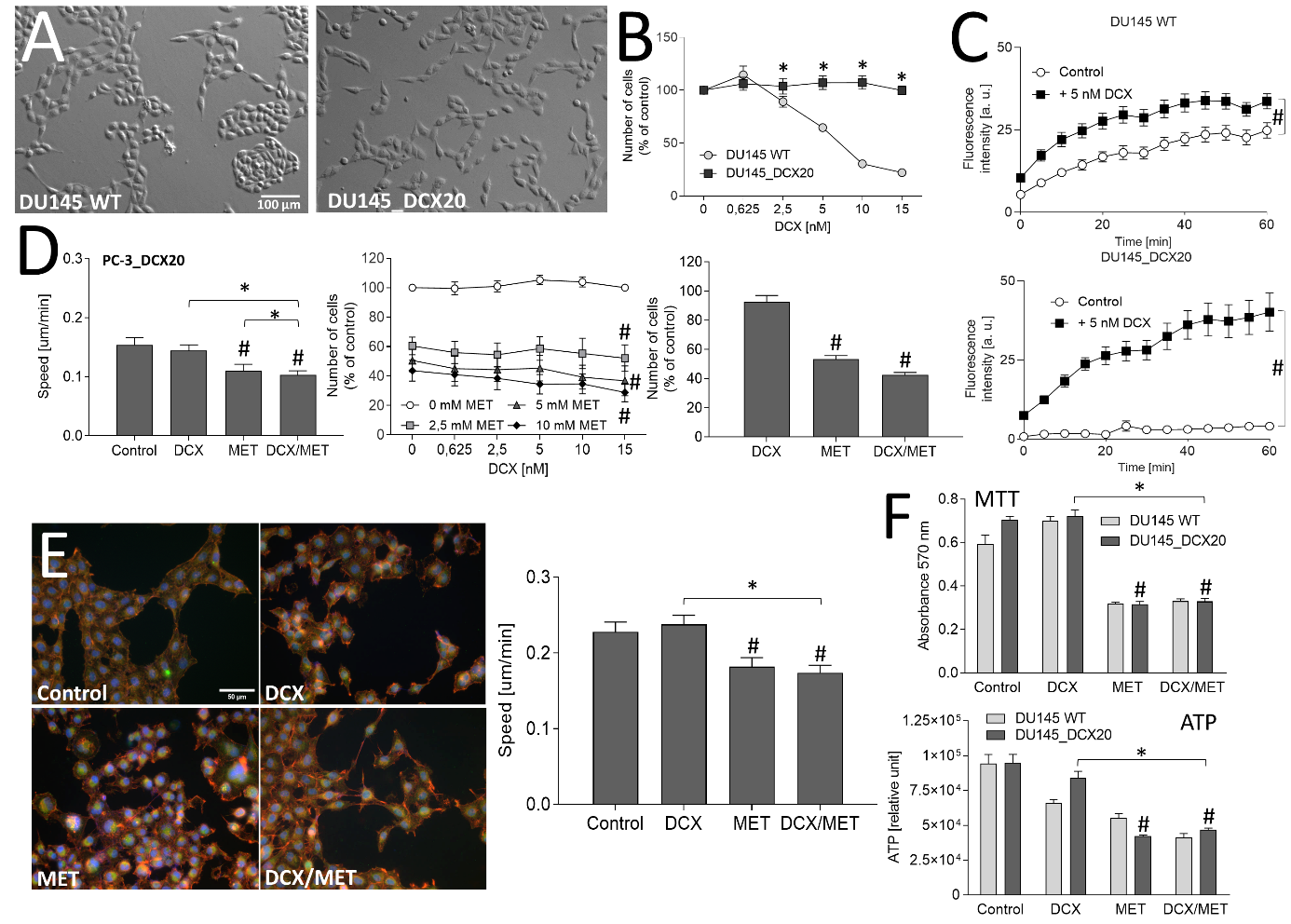
***

**Figure S5. The sensitivity of DU145_DCX20 cells to the combined DCX/MET treatment. (A)** Morphology of DU145 WT and DU145_DCX20 cells in control conditions. **(B)** Effect of DCX (0.625-15 nM) on the proliferation of DU145 WT and DU145_DCX20 cells. **(C)** Drug-efflux efficiency in DCX-treated DU145 WT and DU145_DCX20 cells. **(D)** Effect of the combined DCX/MET treatment on the motility (left) and proliferation of DU145_DCX20 cells (middle, right). **(E)** Actin cytoskeleton architecture (F-actin: red, vinculin: green and DNA: blue) and motility estimated in long-term (48 hours) DCX/MET (2,5 nM/10 mM)-treated DU145_DCX20 cells. **(F)** Viability of DCX/MET-treated DU145_DCX20 populations after the long-term (48 hours) DCX/MET (2.5 nM/10 mM)-treatment estimated with MTT and ATP assay. The statistical significance of the differences was tested with t-Student test (in B, C, proliferation in D, F), or by one-way ANOVA followed by post hoc Tukey's HSD (motility in D, E). #P ≤ 0.05 vs untreated control or 0 mM MET in D (middle); *P ≤ 0.05 as indicated in the charts or vs. DU145 WT (B). All results are representative of at least three independent experiments (N ≥ 3). Error bars represent SEM. **Note a relatively low DCX/MET synergy in DU145_DCX20 populations (cf. Fig. 2).**


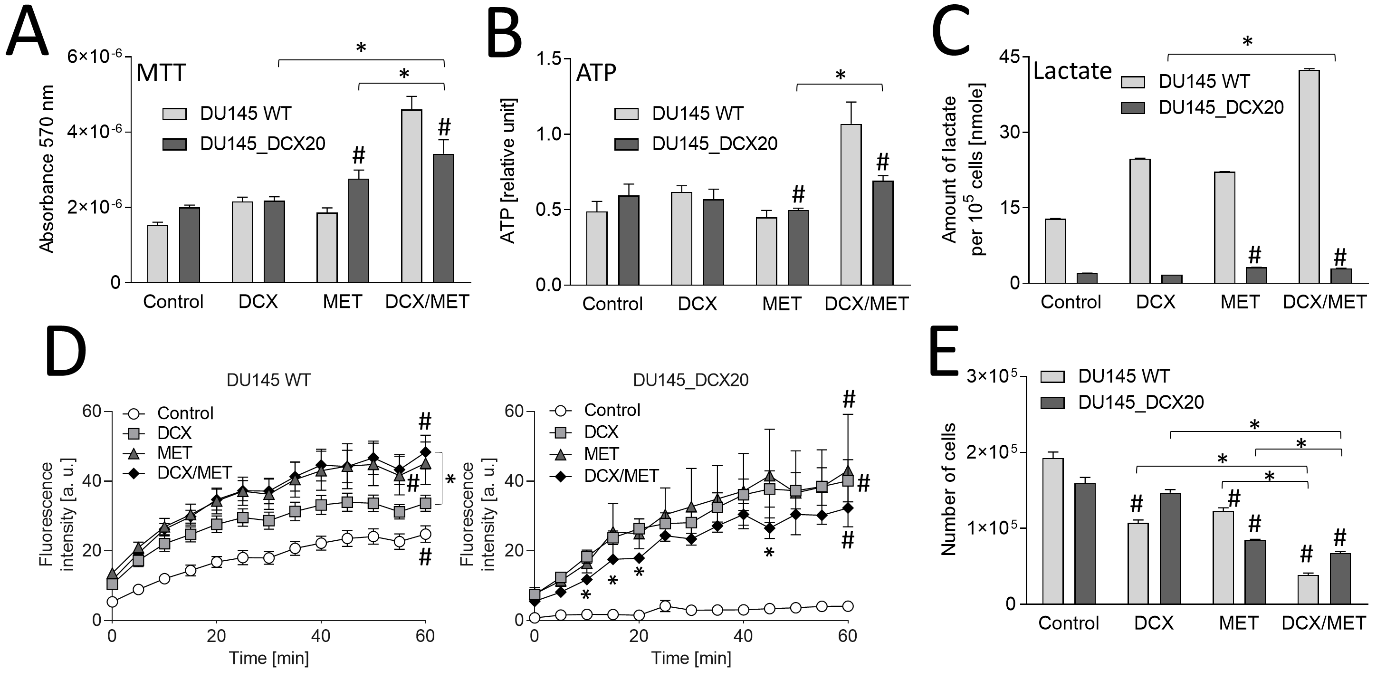


**Figure S6. DCX/MET-induced metabolic decoupling in prostate cancer DU145 populations.** (A-C) DU145 WT and DU145_DCX20 cells were subjected to DCX and/or MET (2.5 nM/10 mM; 48 hours). Metabolic activity (MTT, **A)**, ATP contents **(B)** and lactate production **(C)** was analyzed and calculated per 10^5^ cells. **(D)** Cells were incubated as in A and drug-efflux efficiency was measured with calcein efflux assay. **(E)** Proliferation of DCX/MET-treated DU145 WT and DU145_DCX20 cells. The statistical significance of the differences was tested with t-Student test (in A, B, C, proliferation in D, F), or by one-way ANOVA followed by post hoc Tukey's HSD (motility in D, E). #P ≤ 0.05 vs untreated control; *P ≤ 0.05 as indicated in the charts or vs DCX. All results are representative of a least three independent experiments (N ≥ 3). Error bars represent SEM. **Note a less pronounced metabolic decoupling in DU145_DCX20 populations.**

**
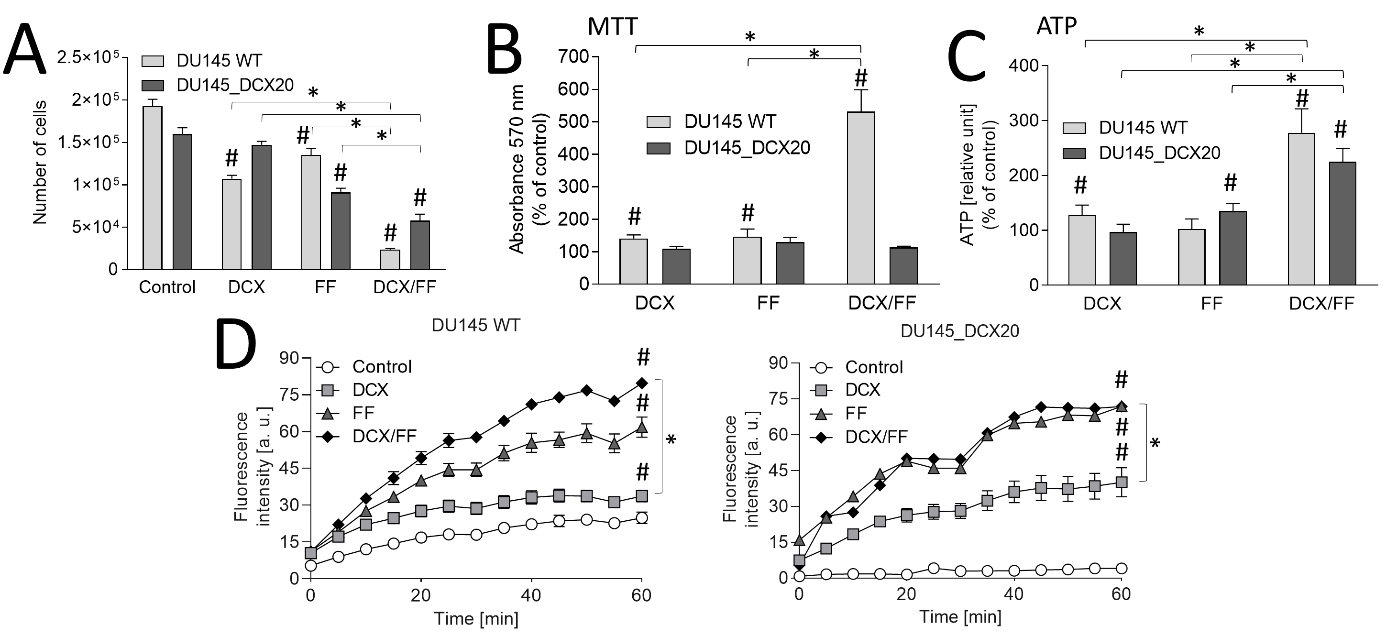
**

**Figure S7. Effect of combined DCX/FF treatment on the metabolic balance in prostate cancer DU145 cells. (A)** Proliferation of DCX/FF treated DU145 WT and DU145_DCX20 cells. DU145 WT and DU145_DCX20 cells were subjected to DCX and/or FF (2.5 nM/25 μM; 48 hours). Metabolic activity MTT **(B**) and ATP contents (**C)** were analyzed and calculated per 10^5^ cells. **(D)** Cells were incubated as in A and drug efflux efficiency was measured with calcein efflux assay. The statistical significance of the differences was tested with t-Student test; #P ≤ 0.05 vs untreated control; *P ≤ 0.05 as indicated in the charts. All results are representative of a least three independent experiments (N ≥ 3). Error bars represent SEM. **Note the metabolic decoupling of DU145 cells.**


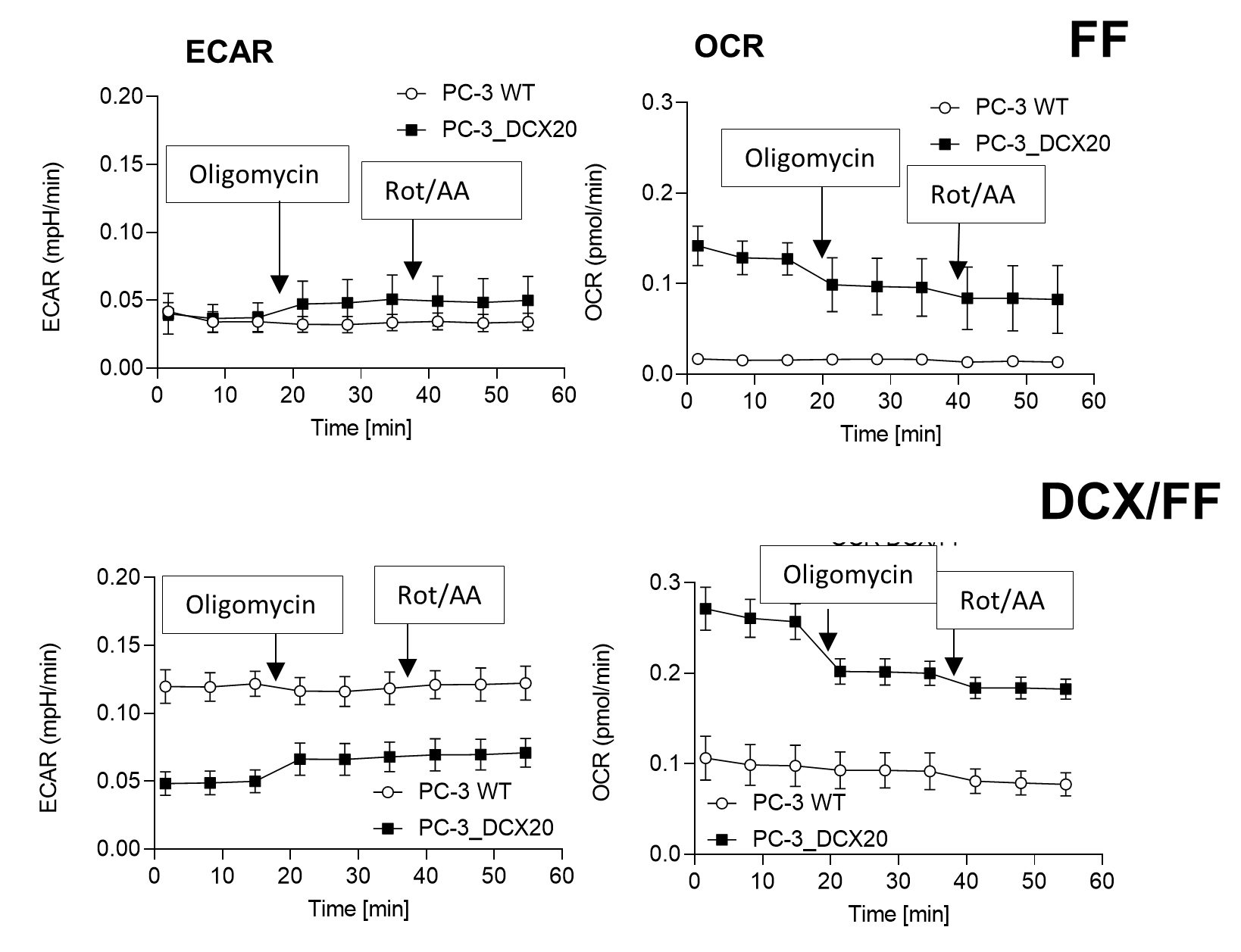


**Figure S8. Metabolic profile of DCX/FF-treated PC-3 WT and PC-3_DCX20 cells.** OCR/ECAR traces of control and DCX/FF-treated (48 hours) PC-3 WT cells and their PC-3_DCX20 counterparts were analyzed with the Seahorse Analyzer XF HS Mini and XFp software, using the Real-Time ATP Rate Assay (arrows show the time-points of oligomycin (1 μM) and rotenone/antimycin A application (1 μM/0.5 μM)). **FF induces incomplete Warburg effect in prostate cancer PC-3 cells.**
