# Supplementary material for: Acquired drug resistance interferes with the susceptibility of prostate cancer cells to metabolic stress
Source: Cell Mol Biol Lett. 2022 Nov 18;27:100. doi: 10.1186/s11658-022-00400-1 (PMC9673456; doi:10.1186/s11658-022-00400-1)

DCX/MET vs. DCX

PC-3 WT

ANOVA & post hoc & FC 1.2	ANOVA & post hoc	SUM	ANOVA & post hoc & FC 1.2	ANOVA & post hoc	SUM	
110	1	111	31		31	142

DNA replication
Cellular response to stress
Chromosome organization
Primary metabolic process

Endoplasmic reticulum

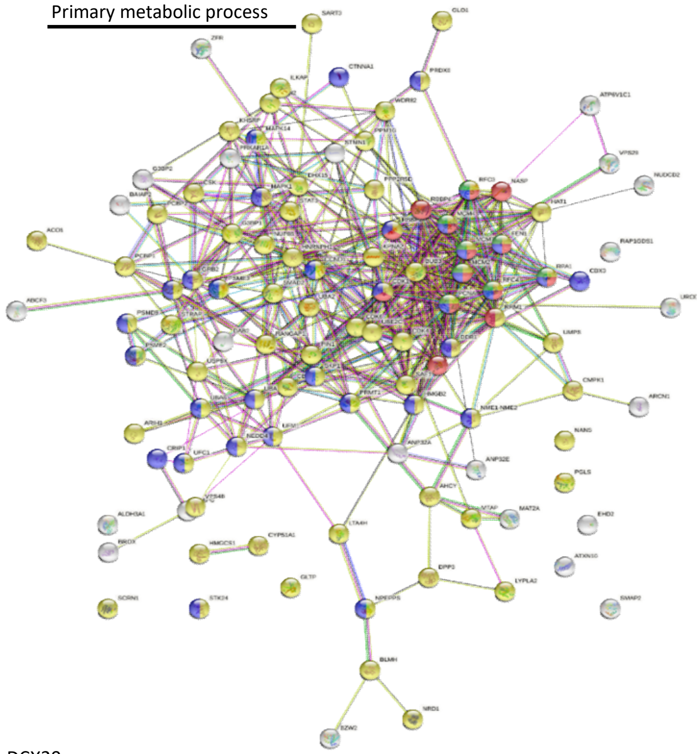

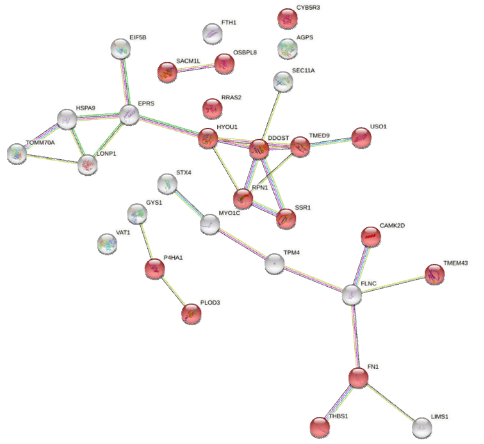

PC-3_DCX20

ANOVA & post hoc & FC 1.2	ANOVA & post hoc	SUM	ANOVA & post hoc & FC 1.2	ANOVA & post hoc	SUM	
44		44	50		50	94

Regulation of catabolic process
G1/S transition of mitotic cell cycle
Negative regulation of mrna metabolic process
Cadherin binding
G1/S Transition
VEGFA-VEGFR2 signaling pathway

Vesicle-mediated transport
Immune system process
Extracellular exosome
Phagosome

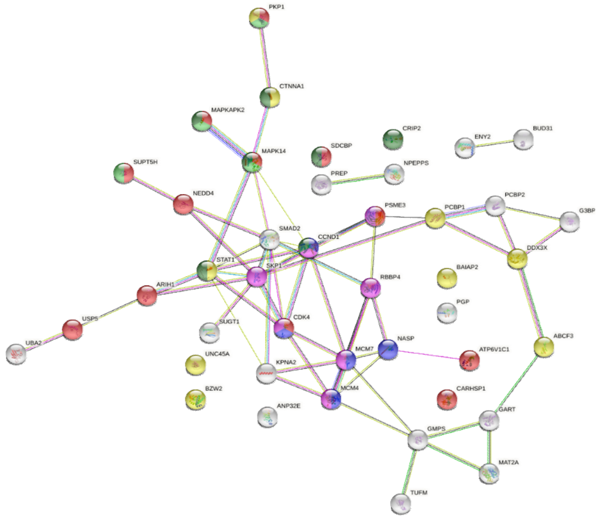

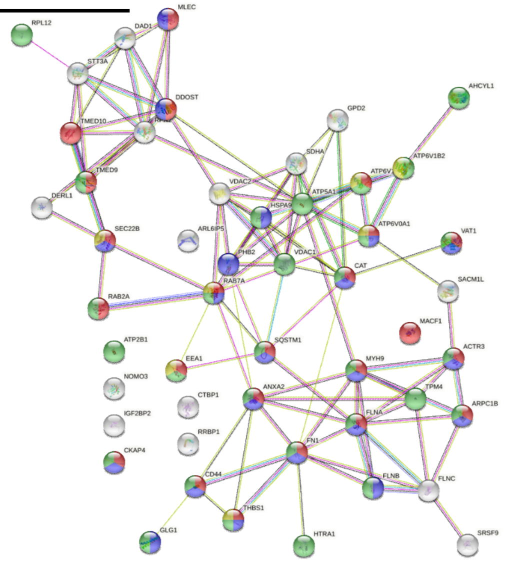

Supplement: Supplementary file 2 — Additional file 2. Appendix 1: Proteomic analyses of DCX/MET-treated PC-3 WT and PC-3_DCX20 cells. [file 11658_2022_400_MOESM2_ESM.pdf]
