# Supplementary material for: Acquired drug resistance interferes with the susceptibility of prostate cancer cells to metabolic stress
Source: Cell Mol Biol Lett. 2022 Nov 18;27:100. doi: 10.1186/s11658-022-00400-1 (PMC9673456; doi:10.1186/s11658-022-00400-1)

DCX/FF vs. DCX

PC-3 WT

ANOVA & post hoc & FC 1.2	ANOVA & post hoc	SUM	ANOVA & post hoc & FC 1.2	ANOVA & post hoc	SUM	
71	1	72	59		59	131

Chromosome organization
G1/S transition of mitotic cell cycle
DNA replication

Vesicle-mediated transport
Transport
Nucleotide binding
Protein processing in endoplasmic reticulum

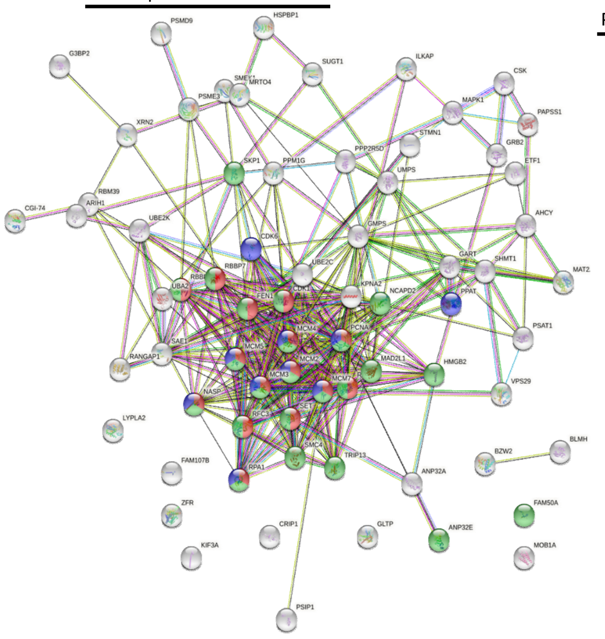

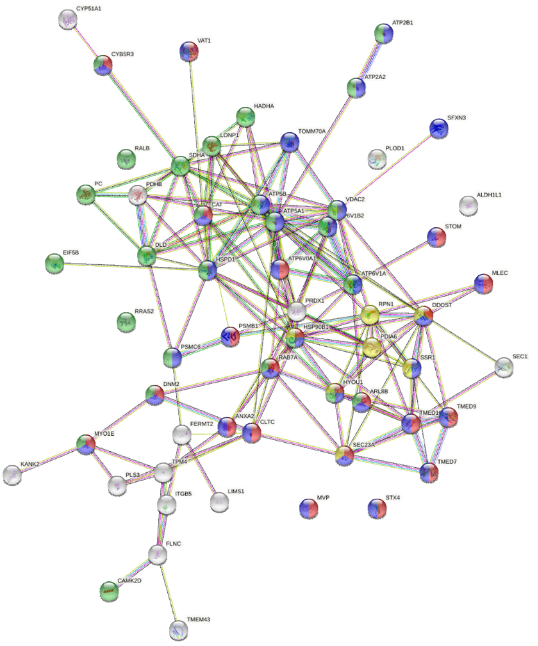

PC-3_DCX20

ANOVA & post hoc & FC 1.2	ANOVA & post hoc	SUM	ANOVA & post hoc & FC 1.2	ANOVA & post hoc	SUM	
19		19	42		42	61

Mitotic cell cycle phase transition
Chromatin organization
G1/S Transition

Vesicle-mediated transport
Immune effector process
Phagosome acidification
Immune system process

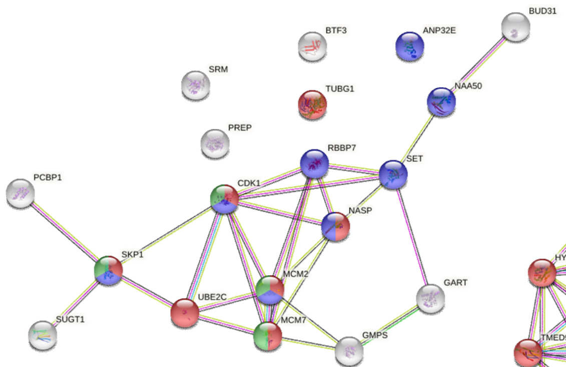

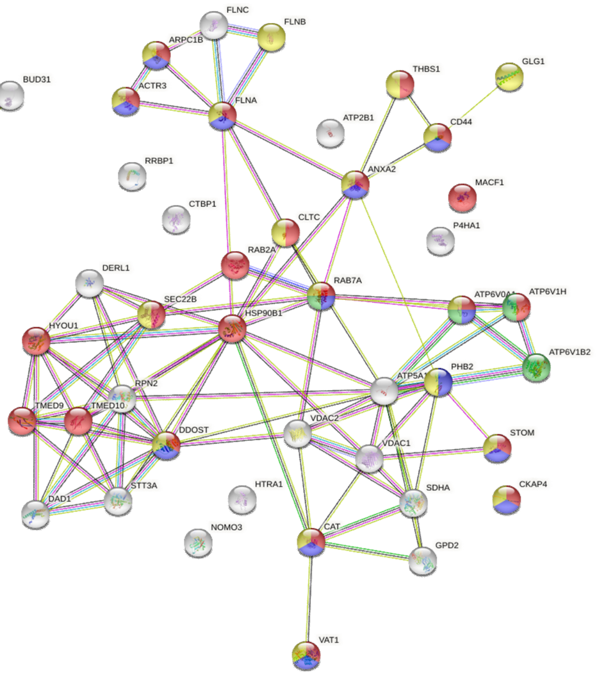

Supplement: Supplementary file 3 — Additional file 3. Appendix 2: Proteomic analyses of DCX/FF-treated PC-3 WT and PC-3_DCX20 cells. [file 11658_2022_400_MOESM3_ESM.pdf]
